# Identification of a CSC-Associated miRNA Signature in NSCLC and Functional Characterization of hsa-let-7a-3p

**DOI:** 10.3390/biomedicines14081737

**Published:** 2026-07-31

**Authors:** Ángela Y. García Fonseca, Carlos Javier Alméciga-Díaz, Andrés F. Aristizábal-Pachón

**Affiliations:** 1 Departamento de Nutrición y Bioquímica, Faculty of Science, Pontificia Universidad Javeriana, Bogotá DC 110231, Colombia; angela_garcia@javeriana.edu.co; 2 Institute for the Study of Inborn Errors of Metabolism, Faculty of Science, Pontificia Universidad Javeriana, Bogotá DC 110231, Colombia; cjalmeciga@javeriana.edu.co; 3 Foundation for Applied Clinical and Molecular Applied Cancer Research—FICMAC, Bogotá DC 110111, Colombia

**Keywords:** non-small cell lung cancer, cancer stem cells, metastasis, epithelial-to-mesenchymal transition, miRNAs

## Abstract

**Background**: Metastasis is the leading cause of mortality in lung cancer and is regulated by multiple molecular mechanisms, including microRNAs (miRNAs). Although cancer stem cells (CSCs) and epithelial–mesenchymal transition (EMT) contribute to metastatic progression, the miRNA networks underlying these phenotypes remain poorly characterized in non-small cell lung cancer (NSCLC). **Aim**: To identify miRNA signatures associated with CSCs and EMT in NSCLC and functionally characterize hsa-let-7a-3p. **Methods**: EMT was induced in A549 and NCI-H1975 cells by dCas9-mediated activation of TWIST, whereas CSC-enriched populations were generated by CD133-based sorting and stem cell culture conditions. Small RNA sequencing, bioinformatic analyses, qPCR validation, and functional assays were performed to identify and characterize phenotype-associated miRNAs. **Results**: Small RNA sequencing identified distinct miRNA expression profiles associated with EMT and CSC enrichment. Comparative analysis identified 13 commonly downregulated and 13 commonly upregulated miRNAs shared by CSCs from A549 and H1975 cells, suggesting conserved post-transcriptional regulatory mechanisms. Functional enrichment and miRNA–target interaction network analyses linked the miRNA signatures to pathways involved in epithelial plasticity, stemness, and tumor progression, including Wnt, TGF-β, mTOR, focal adhesion, adherens junction, and regulation of the actin cytoskeleton. Among the dysregulated miRNAs, hsa-let-7a-3p was consistently upregulated in CD133^+^ CSC-enriched cells from both NSCLC cell lines. Functional assays showed that hsa-let-7a-3p overexpression significantly reduced clonogenic capacity and showed a trend toward decreased invasion without affecting proliferation. **Conclusion**: This study identifies miRNA signatures associated with CSC-enriched and EMT-associated phenotypes in NSCLC and demonstrates that these signatures represent coordinated post-transcriptional regulatory programs involved in epithelial plasticity, stemness, and metastatic progression. Functional validation of hsa-let-7a-3p further supports its role as a context-dependent regulator of CSC biology and highlights the potential of miRNA signatures as diagnostic biomarkers in NSCLC.

## 1. Introduction

Non-small cell lung cancer (NSCLC) is a highly aggressive tumor that causes metastasis in most cases, leading to a significant number of deaths worldwide [1]. According to the World Health Organization (WHO), lung cancer accounted for 1,817,469 deaths globally in 2022, making it the leading cause of cancer-related mortality. Among these cases, approximately 80–85% were attributed to the non-small cell lung cancer (NSCLC) subtype, highlighting its critical role in global cancer burden [2]. Metastasis is the main driver of death in NSCLC, with reports indicating that approximately 90% of patients with metastasis ultimately die [3,4].

It is now understood that, during metastatic progression, tumor cells undergo various molecular and phenotypic changes to acquire motility and invasiveness [4]. A key process in this transformation is the epithelial-to-mesenchymal transition (EMT), which depends on the activation of several genes, including transcription factors such as Twist, Nanog, and Snail, among others [5]. EMT transforms epithelial tumor cells into a mesenchymal phenotype characterized by the loss of epithelial markers, including E-cadherin, cytokeratins, occludin, and claudins, together with the acquisition of mesenchymal markers such as N-cadherin, vimentin, fibronectin, and ZEB1 [6,7]. Beyond this transition, a subset of mesenchymal-like cells acquires enhanced self-renewal and tumor-initiating capacities and expresses stem cell-associated markers, including CD133, CD44, aldehyde dehydrogenase (ALDH), epithelial cell adhesion molecule (EpCAM), and ATP-binding cassette transporter G2 (ABCG2) [8].

The existence of cancer stem cells (CSCs) plays a pivotal role in metastasis, enabling the formation of secondary tumors, resistance to conventional treatments, and recurrence [9,10]. In recent years, increasing evidence suggests that, in addition to the dysregulation of protein-coding genes, alterations in the expression of non-coding RNAs, particularly microRNAs (miRNAs), contribute to both EMT and CSC generation [11,12,13]. Although numerous studies have characterized dysregulated miRNA signatures in NSCLC by comparing tumor tissues with adjacent normal tissues or non-tumor controls [14,15,16], considerably less is known about miRNA signatures associated with distinct cellular subpopulations that coexist within tumors. Comparative analyses of miRNA expression in CSCs and EMT-associated cell populations derived from NSCLC models remain scarce. Consequently, the extent to which these aggressive phenotypes share or differ in their post-transcriptional regulatory programs has not been systematically explored.

Therefore, the aim of this study was to identify dysregulated miRNA signatures associated with EMT and CD133^+^ CSC-enriched populations in NSCLC, and compare the regulatory programs underlying these two metastatic phenotypes. By characterizing miRNA signatures involved in tumor and CSC biology in NSCLC, it becomes possible to identify coordinated regulatory programs rather than isolated molecular alterations. Since miRNAs function cooperatively to modulate multiple target genes and signaling pathways, defining these signatures may provide valuable insights into the mechanisms underlying colonization, invasion, and metastatic progression, while facilitating the identification of robust biomarker panels and novel diagnostic targets.

To achieve this, we obtained mesenchymal-like and CD133^+^ CSC-enriched cells from the NSCLC cell lines A549 and NCI-H1975 (H1975). RNA was extracted from these populations and sequenced using small RNA-seq, followed by analysis to determine the differential expression profile of miRNAs. From this analysis, we identified a distinct CSC-associated miRNA signature, and one of the identified candidate miRNAs, hsa-let-7a-3p, was selected for detailed functional analysis.

## 2. Materials and Methods

### 2.1. Epithelial–Mesenchymal Transition from Lung Cancer Cell Line

Two lung cancer cell lines, A549 and NCI-H1975 (H1975), were used in this study, which were obtained from ATCC (Manassas, VA, USA) and are NSCLC adenocarcinomas. The A549 cell line is derived from the lung of a 58-year-old Caucasian male smoker, while the H1975 cell line was isolated in 1988 from the lungs of a nonsmoking woman. A549 and NCI-H1975 cell lines were selected as complementary models of non-small cell lung cancer (NSCLC) to evaluate EMT induction and the acquisition of cancer stem cell-associated phenotypes across distinct molecular backgrounds, A549 cells harbor KRAS mutations and are widely used as a model for studying EMT, cellular plasticity, and tumor progression. In contrast, NCI-H1975 cells carry the clinically relevant EGFR L858R and T790M mutations, which are associated with resistance to EGFR targeted therapies and have been linked to tumor heterogeneity, epithelial–mesenchymal plasticity, and stem-like characteristics. The inclusion of these two cell lines allowed the evaluation of TWIST-mediated EMT induction in molecularly distinct NSCLC contexts. Thereby, enabling the assessment of whether the acquisition of EMT- and CSC-associated traits occurs in a consistent manner across different genetic backgrounds. Given the recognized heterogeneity of NSCLC, the use of both models was intended to provide a broader representation of the biological diversity observed in lung adenocarcinoma and to identify potential context-dependent differences in the regulation of EMT and stemness programs.

The cells were cultured in DMEM with 10% FBS until they reached confluence. After that, EMT was induced by transduction with N-twist Lentiviral Activation Particles (human) (sc-415166-LAC, Santa Cruz Biotechnology, Dallas, TX, USA) [17], which is a lentiviral-based vector used for activating the expression of Twist in human cells. The N-twist Lentiviral Activation Particles (h) include the following synergistic activation mediator (SAM): a deactivated Cas9 (dCas9) nuclease, which is fused to the VP64 transactivation domain, an MS2-p65-HSF1 fusion protein, and a 20-nucleotide gRNA targeted to 200–250 nucleotides upstream of the transcriptional start site of Twist. These particles also carry resistance genes for blasticidin, hygromycin, or puromycin to select transduced colonies. Transduction and clone selection were carried out following the manufacturer’s instructions. copGFP Lentiviral Particles (sc-108084) was used as a control for measuring transduction efficiency.

### 2.2. Generation of Spheroids Derived-Cancer Stem Cells from Lung Cancer Cell Line

The cells transduced with N-twist Lentiviral Particles, were expanded them and potential cells with a cancer stem cell (CSC) phenotype were isolated using the CD133 MicroBead Kit (catalog #130-097-049, Miltenyi Biotec, Bergisch Gladbach, Germany) [18,19], following the manufacturer’s instructions. Briefly, cells were labeled with CD133 MicroBeads and separated using a MACS column under a magnetic field. The CD133^+^ cells are retained in the column, while unlabeled cells are washed away. Finally, the CD133^+^ cells were eluted by removing the column from the magnetic field, collecting the purified cells in a new tube. Isolated cells were seeded in ultra-low attached 24-well plate with DMEM/F12 medium free of fetal bovine serum (FBS), supplemented with 2 mM glutamine, 20 ng/mL Epidermal Growth Factor (EGF), 10 ng/mL β-Fibroblast Growth Factor (bFGF), 1X Insulin-Transferrin-Selenium (ITS), and 1X B27, to stimulate the generation of CD133^+^ CSC-enriched cells.

### 2.3. RNA Isolation

RNA isolation was performed from four populations for each cell line: cells without treatment, cells transduced with GFP as control transduction, cells transduced with Twist, and CD133^+^ CSC-enriched cells, for both A549 and H1975 cell lines. RNA was extracted using the Quick-DNA/RNA™ Miniprep Kit (Catalog #D7001, Zymo Research, Irvine, CA, USA). RNA concentration for each sample was confirmed using a Nanodrop, and the quality was measured with the Qubit™ RNA IQ Assay Kits (Catalog #Q33221, Thermo Fisher, Waltham, MA, USA). 

### 2.4. Flow Cytometry 

For flow cytometry analysis of TWIST protein detection, A549 and H1975 cells were trypsinized and collected. One million cells per condition were transferred to microcentrifuge tubes and centrifuged at 1800 rpm for 5 min. After removing the supernatant, cells were washed once with 1X PBS and centrifuged again. Cells were then resuspended in 1 mL of fixation/permeabilization solution composed of 300 µL of medium DMEM and 700 µL of absolute methanol, added dropwise while gently mixing. Samples were incubated for 30 min at −20 °C, then centrifuged at 5000 rpm for 5 min, and the supernatant was discarded by inversion. Cells were washed with 500 µL of 1X PBS containing 2% BSA (blocking buffer) and centrifuged again. Subsequently, cells were incubated overnight at 4 °C with 10 µL of anti-TWIST primary antibody (Twist2C1a, sc-81417, 100 µg/mL; Santa Cruz Biotechnology, Dallas, TX, USA) diluted in 290 µL of blocking buffer. After incubation, cells were centrifuged at 5000 rpm for 5 min, and cells were washed twice with blocking buffer. A second blocking step was performed by incubating the cells in 500 µL of blocking buffer for 30 min at 4 °C, followed by centrifugation. An Alexa Fluor 488-conjugated anti-mouse IgG (Catalog # A32723, Thermo Fisher Scientific, Waltham, MA, USA) was added at a 1:400 dilution and the samples were incubated for 1 h at 4 °C in the dark. Cells were washed twice with blocking buffer, resuspended in 300 µL of 1X PBS, and analyzed on a Guava flow cytometer (Merck Millipore, Darmstadt, Germany). Proper controls including unstained cells and secondary antibody-only samples were used for gating.

### 2.5. Evaluation of Markers for Each Phenotype Was Conducted by qPCR

The level of various markers, known for each phenotype, were evaluated in the four cell populations using qPCR. From the RNA, cDNA was synthesized using OneScript^®^ Plus Reverse Transcriptase (Cat. No. G237, Applied Biological Materials Richmond, Vancouver, BC, Canada). Briefly, a reaction mixture was prepared with 4 µL of 5X RT buffer, 1 µL of dNTPs, 1 µL Oligo (dT), 1 µL of OneScript^®^ Plus RTase, and 20 ng/µL RNA. The mixture was incubated for 15 min at 50–55 °C, followed by heating at 85 °C for 5 min. Next, qPCR was carried out using BlasTaq™ 2X PCR Master Mix (Cat. No. G890, Applied Biological Materials, Richmond, Vancouver, BC, Canada). The reaction mix was prepared with 5 µL 2X BlasTaq™ PCR Master Mix, 10 μM each primer (see Supplementary Table S1 for sequences), and 1 µL template DNA. The protocol used on the thermocycler was as follows: Enzyme activation at 95 °C for 3 min, denaturation at 95 °C for 15 s, and annealing/extension at 60 °C for 1 min. These steps were repeated for 40 cycles, followed by a melting curve from 60 °C to 90 °C, with a 5 °C increase every 5 s. The qPCR was performed using a CFX96 Touch real-time thermal cycler (BioRad, Hercules, CA, USA). The results were analyzed using the Schefe method [20] and gene quantification was reported as the geometric averaging of multiple internal control genes [21] reporting the geometric mean considering the two reference genes, GAPDH and RPL27. Expression data are presented as the geometric mean of relative expression ratio (rER), obtained from independent biological replicates.

### 2.6. Small RNA Sequencing

Small RNA libraries were prepared and sequenced by Novogene using next-generation sequencing (NGS) technology on an Illumina NovaSeq 6000 platform, following the manufacturer’s protocol with a single-end 50 bp (SE50) sequencing approach. A minimum of 10 million raw reads per sample was targeted. For this analysis, three biological replicates of CD133^+^ CSC-enriched cells, cells transduced with Twist, cells transduced with GFP, and adherent parental cells were sequenced for each cell line.

### 2.7. Differential Expression of miRNAs

Data processing was carried out using the Galaxy platform, specifically utilizing the Genomic File Manipulation tool [22]. Briefly, the raw data were assessed in FASTQ format, and adapter sequences and nonspecific dimers were removed using the Cutadapt tool. Reads were aligned to the human genome using MiRDeep2 Mapper and identified miRNAs based on precursor and mature miRNAs listed in miRbase [23]. Next, the MiRDeep2 Quantifier tool was employed to quantify the abundance of these miRNAs in each sample.

Based on RNA-seq data, differential expression analysis of miRNAs was performed by establishing distinct comparison groups for each cell line: (1) non-treatment cells vs. cells transduced with GFP as control, (2) cells transduced with GFP vs. cells transduced with Twist, and (3) cells transduced with Twist vs. CD133^+^ CSC-enriched cells (CSCs). To achieve this, the DESeq2 tool was used. Then, the Filter tool was used to generate a list containing only the miRNAs with an adjusted *p*-value < 0.05. From these results, dysregulated miRNAs were categorized for each comparison group into two separate lists, including either upregulated or downregulated miRNAs. 

To identify miRNAs shared between comparison groups within each cell line, a Venn diagram was generated using the Bioinformatics & Evolutionary Genomics tool (refer to Bioinformatics & Evolutionary Genomics Venn Tool). Three input files were provided: Input 1 contained the list of dysregulated miRNAs from non-treated cells versus GFP-expressing cells; Input 2 included dysregulated miRNAs from GFP versus Twist-expressing cells; and Input 3 included those from Twist-expressing cells versus CD133^+^ CSC-enriched cells. miRNAs that were not shared across any of the comparisons were considered uniquely dysregulated and selected for further analysis. Subsequently, the uniquely dysregulated miRNAs identified for each condition in the A549 and H1975 cell lines were compared using a Venn diagram. The overlapping miRNAs were considered the most relevant dysregulated candidates, and from this list, one miRNA was selected for functional assays.

### 2.8. Targets Identification

To identify high-confidence targets of the hsa-let 7a-3p, we retrieved experimentally validated interactions from the TarBase v9 database [24,25]. This database compiles miRNA–target interactions that have been verified through biological experiments, comprising approximately six million experimentally validated pairs. High-confidence interactions are supported by 15 direct experimental approaches, including reporter assays, CLIP-seq, CLASH, AGO-CLIP, and HITS-CLIP, among others, as well as 22 high-throughput techniques such as microarray, Western blot, qPCR, and related methods [24]. For this study, the following parameters were set: Homo sapiens, high experimental throughput, and just direct experimental type.

### 2.9. Enrichment Analysis

Enrichment analyses were conducted using the Database for Annotation, Visualization, and Integrated Discovery (DAVID) [26]. A list of ENSEMBL gene IDs was uploaded, corresponding to the genes regulated by the miRNAs into the DIANA tool TarBase v9, using the following parameters: Organism = Homo sapiens, Identifier = ENSEMBL_GENE_ID, and List type = Gene list. To prioritize the most robust interactions within this validated set, we further filtered the target genes by applying a microT score threshold of ≥0.9, as provided by the DIANA-microT algorithm, which predicts the likelihood of the interaction. This approach ensured that our downstream analysis focused on a high-confidence list of targets supported by both experimental validation and strong in silico prediction. 

Functional enrichment analysis was performed with DAVID, which applies an empirical sampling-based algorithm supported by multiple databases. For pathway analysis, we focused on KEGG-annotated data, while Gene Ontology (GO) terms were used to assess biological processes, cellular components, and molecular functions. Enrichment terms with an adjusted *p*-value ≤ 0.05 and relevance to CSC maintenance were subsequently selected for further interpretation.

Also, an enrichment analysis using DIANA-miRPath v.4.0 [27] was conducted with the same list of genes selected previously, under the following conditions: Targets: TarBase v8.0 (which includes only experimentally validated miRNA-target interactions), TarBase targets: Direct, Species: Homo sapiens, miRNA Annotation: miRBase v22.1, Terms/Pathways: KEGG, Merging Method: Pathways union, Testing Method: Classic analysis, *p*-value threshold: 0.05, FDR Correction.

### 2.10. miRNA–Target Network and Functional Enrichment Analysis

The miRNA–target interaction network was generated using miRNet. Homo sapiens was selected as the reference organism, and experimentally validated miRNA–target interactions were retrieved from the TarBase and miRTarBase databases using miRBase IDs as input. To prioritize highly connected target genes while retaining all input miRNAs, the network was filtered using the “All but miRNA nodes” option with a degree cutoff ≥ 5. The filtered gene set was subsequently subjected to Kyoto Encyclopedia of Genes and Genomes (KEGG) pathway analyses and functional enrichment analysis using Gene Ontology (GO) Biological Process [28]. Signaling pathways associated and enriched biological processes with epithelial–mesenchymal transition (EMT) and cancer stem cell (CSC) biology were identified and interpreted.

### 2.11. Validation hsa-let 7a-3p Detection by qPCR

microRNA levels were analyzed with the stem-loop RT method [29]. Total RNA was extracted from each sample using TRIzol reagent (Cat. No. 15596026, Invitrogen, Carlsbad, CA, USA) following the manufacturer’s instructions. RNA concentration was determined with a NanoDrop TM 2000 Spectrophotometer (Thermo Fisher, Waltham, MA, USA). The cDNA was synthesized using the OneScript^®^ Plus cDNA Synthesis Kit (Cat. No. G237Applied Biological Materials, Vancouver, BC, Canada), with 20 ng RNA and a specific RT primer for hsa-miR-let-7a-3p, part of the TaqMan^®^ MicroRNA Assays ID 002307 (Thermo Fisher Scientific, Waltham, MA, USA). This kit includes specific forward and reverse primers along with a TaqMan^®^ FAM probe, which was used for qPCR in conjunction with the Luna Universal Probe qPCR Master Mix (New England Biolabs, Ipswich, MA, USA). The protocol used for qPCR was as follows: Enzyme activation at 95 °C for 60 s, denaturation at 95 °C for 15 s, and annealing/extension at 60 °C for 30 s, the steps were repeated for 40 cycles. The qPCR was performed using a CFX96 Touch real-time thermal cycler (BioRad, Hercules, CA, USA). Results were analyzed using the Schefé method [15]. The analysis focused on the detection of the presence or absence of microRNAs. For normalization, the non-coding RNA U24 was used.

### 2.12. Transfection and Inhibition of hsa-miR-let-7a-3p

To determine the role of hsa-miR-let-7a-3p in lung cancer cell lines, the cells were transfected with a chemically synthesized miRNA mimic to increase its intracellular levels (Catalog # MCH01002, Applied Biological Materials, Vancouver, BC, Canada) in non-treatment A549 and H1975 cells. The inhibition was performed with mirVana™ miRNA Inhibitor (Catalog # 4464084, Thermo Fisher Scientific, Waltham, MA, USA), which is a single-stranded RNA molecule designed to specifically bind and inhibit the mature miRNA of interest, thereby blocking its interaction with target mRNAs and preventing translational repression or mRNA degradation. A scrambled sequence (Cat. No.: MCH00000, Applied Biological Materials, Vancouver, BC, Canada) was used as a negative control. For each cell line, five conditions were evaluated: (1) non-transfected cells (control), (2) cells transfected with scramble, (3) cells transfected with mimic, (4) cells transfected with mimic + inhibitor, and (5) cells transfected with inhibitor. 

The mimic and inhibitor were transfected with Lipofectamine 3000 Transfection Reagent (Cat. No. L3000015 Thermo Fisher Scientific, Waltham, MA, USA), following the manufacturer’s instructions, with 10 μM mimic or 20 μM inhibitor. The cells were seeded in 6-well plates at a density of 5 × 10^5^ cells per well in Opti-MEM^®^. Subsequently, mimic or inhibitor-lipid complex mixture was added to each well. The transfected cultures were incubated under standard conditions for 24 h. After this time, the medium was removed, and the transfected cells were washed with 1X PBS and added DMEM supplemented with 10% FBS. After 24 h incubation, the cells were trypsinized and divided into for fractions: one fraction to confirm overexpression and inhibition through qPCR as explained above, a second fraction for the proliferation assay, third fraction for clonogenia assay, and the last one for the invasion assay. Three independent experiments were performed in triplicate.

### 2.13. Proliferation Assay 

The proliferation rate was analyzed at several time points: 0, 24 h, 48 h, 72 h, and 96 h using the CyQuant^®^ Direct Proliferation Assay Kit (No. C35011, Thermo Fisher Scientific, Waltham, MA, USA), following the manufacturer’s instructions. The analysis was performed as follows: 48 h after transfection, cells from each condition were seeded at a density of 2500 cells per well in triplicate in 96-well plates. For each time point, independent plates were incubated under standard conditions. At the end of each incubation period, staining with CyQuant^®^ Direct nucleic acid stain was performed according to the manufacturer’s instructions. Briefly, CyQuant^®^ Direct nucleic acid stain was added to each well and incubated for 1 h. The fluorescence intensity is directly proportional to the amount of nucleic acids and thus the number of proliferating cells. The fluorescence emitted was measured using the FLUOstar Omega device (BMG LABTECH, Ortenberg, Germany) with a 480 nm/520 nm excitation/emission filter. The fluorescence values from the triplicate assays were averaged and then normalized with respect to the control cells for each time point evaluated.

### 2.14. Invasion Assay

The Cell Invasion Assay (Basement Membrane), 96-well, 8 µm (ab235697, Abcam, Cambridge, UK), was performed after the transfection period, following the manufacturer’s instructions. Briefly, the day before the experiment, 40 µL of Matrigel was added to each well in the upper chamber, and the plate was stored at 4 °C overnight. On the next day, transfected cells were detached, and 8000 cells were seeded into the top chamber of each well using FBS-free medium. The bottom chamber was filled with DMEM supplemented with 10% FBS to serve as a chemoattractant. The plate was then incubated under standard conditions (37 °C, 5% CO_2_) for 24 h. After incubation, the medium from the bottom chamber was carefully removed and replaced with a mixture of 10 µL Cell Invasion Dye and 90 µL Cell Dissociation Solution per well. Non-invading cells remaining on the upper side of the top membrane were removed with a cotton swab. The cells that had invaded through the membrane and adhered to the lower side were transferred into the bottom chamber using the mixed solution (Dye + Dissociation Solution) and incubated at 37 °C for 60 min. After incubation, the chambers were disassembled, and the top chambers were discarded. The fluorescence in the bottom wells was read at Ex/Em = 530/590 nm using a FLUOstar Omega plate reader (BMG LABTECH, Ortenberg, Germany). Fluorescence values from triplicate wells were averaged and normalized against control cells. A calibration curve was generated prior to the experiment to interpolate the fluorescence values and estimate the number of cells that invaded, based on the equation of the line.

### 2.15. Clonogenic Assay

After transfection, 500 cells from each condition were seeded in a 6-well plate using DMEM supplemented with 10% FBS. The cells were cultivated for 10 days to allow colony formation. A colony was defined as a cluster of at least 50 cells. After this period, the medium was removed, and the cells were fixed with 4% formaldehyde for 2 min, followed by methanol treatment for 15 min. Finally, the cells were stained with Giemsa for 15 min. Colonies were counted using a stereoscope. The clonogenic assay was performed exclusively in the A549 cell line. This decision was based on preliminary observations indicating that A549 cells exhibit a significantly higher and more reproducible plating efficiency under the experimental conditions tested, which is essential for obtaining reliable and statistically powered quantitative data from this assay. H1975 cells did not meet this criterion, and thus were excluded from this functional analysis.

### 2.16. Statistical Analyses

All statistical analyses were carried out using GraphPad Prism 8 (GraphPad Software, San Diego, CA, USA). Data obtained from qPCR, proliferation, invasion, clonogenic, and flow cytometry assays are presented as mean ± standard deviation (SD) from at least three independent biological replicates, each performed with their corresponding technical replicates unless otherwise indicated. Relative expression ratios (rERs) for qPCR analyses were calculated using the Scheffé’s Gene Expression Difference (GED) method, considering the amplification efficiency of each target gene. GAPDH and RPL27 were used as endogenous controls for mRNA analyses, while U24 was used for miRNA normalization.

Statistical differences between groups were evaluated according to the distribution of the data. Comparisons among multiple groups were performed using one-way ANOVA followed by Dunnett’s multiple comparisons test, using the control group as the reference condition. Proliferation assays, which included multiple time points and treatment conditions, were analyzed using two-way ANOVA. When data did not meet parametric assumptions, the Kruskal–Wallis test was used. Differences were considered statistically significant at *p* < 0.05. Significance levels are indicated as follows: *p* < 0.05 (*), *p* < 0.01 (**), *p* < 0.001 (***) and *p* < 0.0001 (****), whereas “ns” indicates no statistically significant difference.

## 3. Results

### 3.1. TWIST Overexpression Induces EMT-Associated Molecular Changes in A549 Cells 

**Figure 1:**
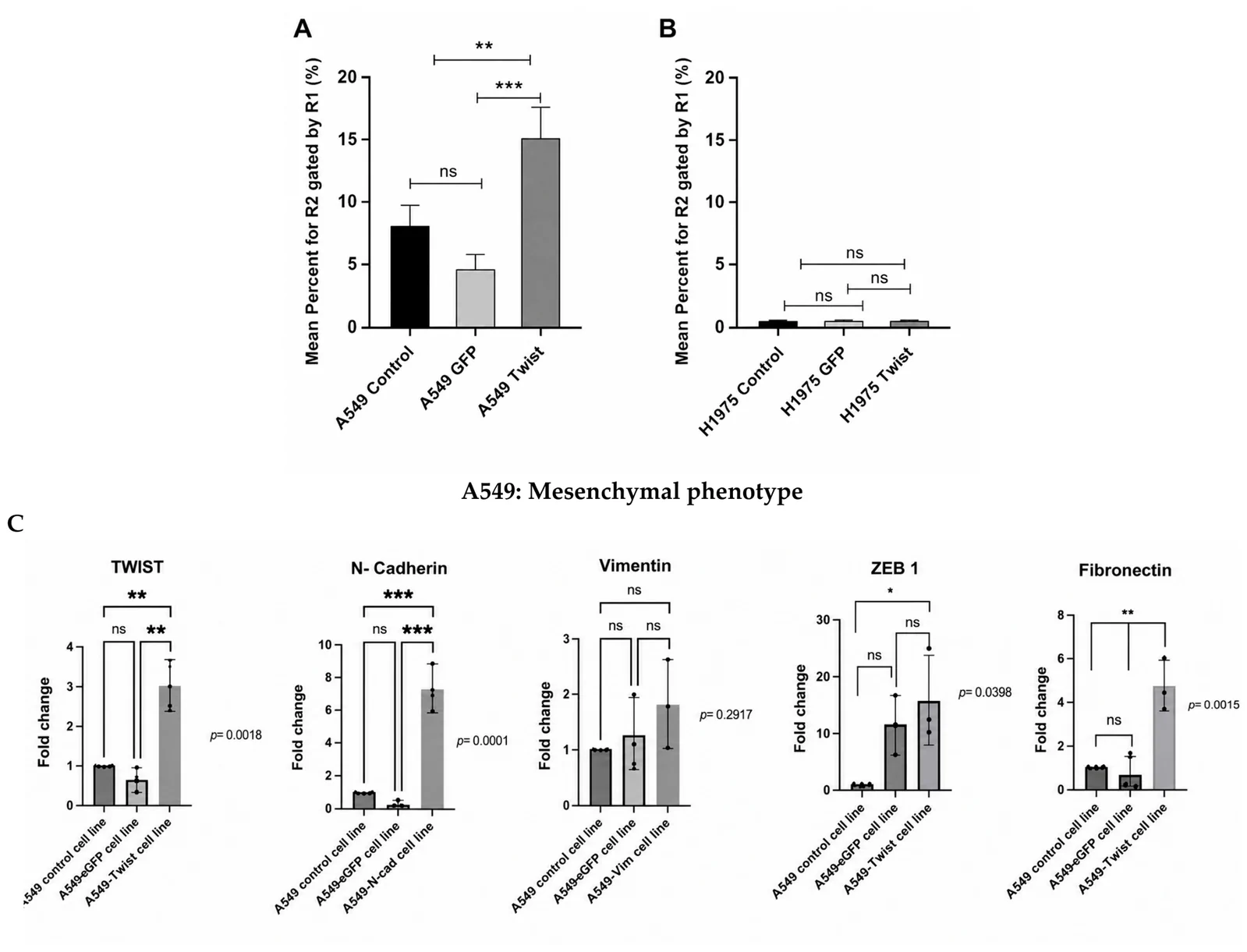

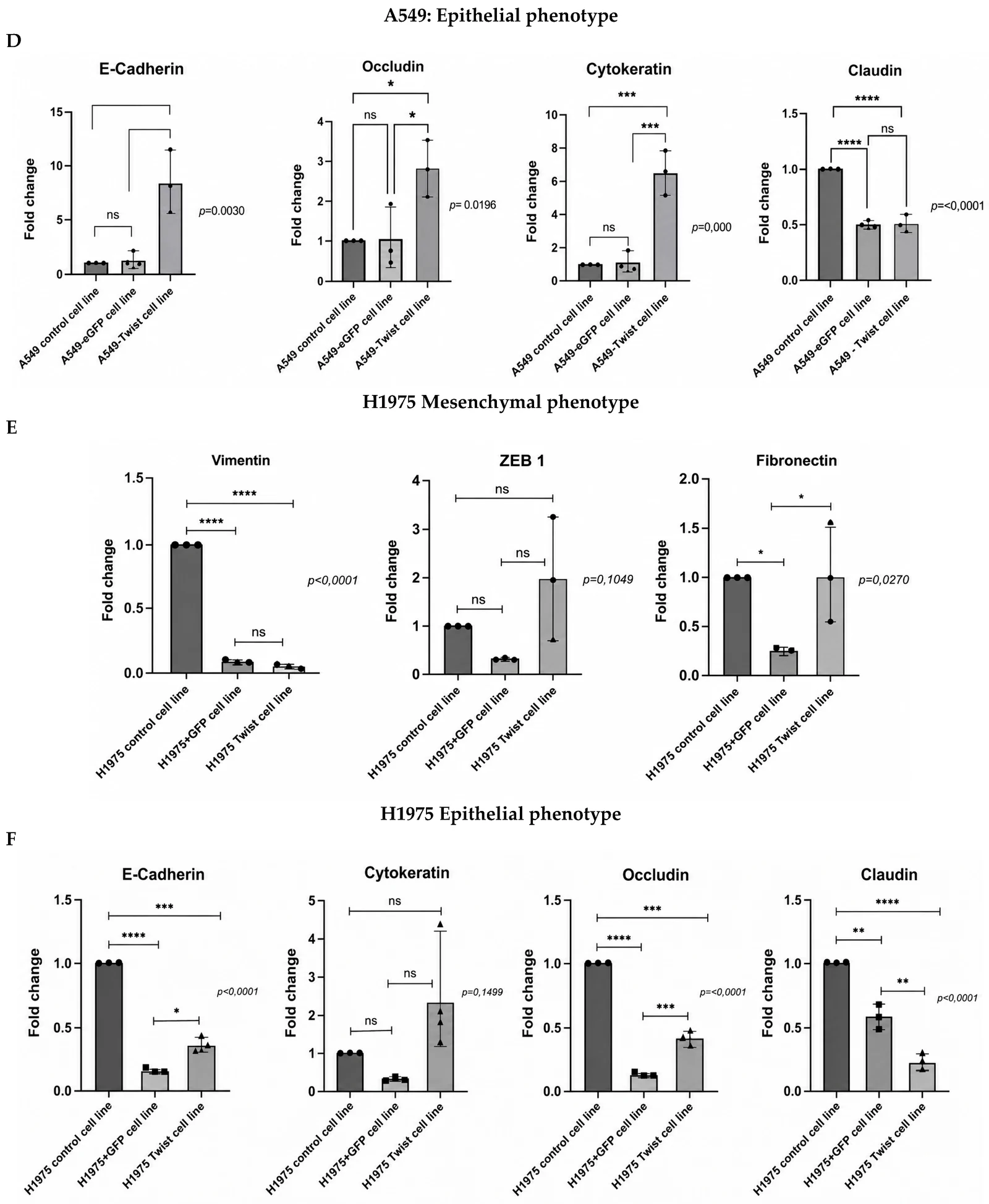
EMT markers evaluated in transduced lung cancer cell lines. To confirm Twist upregulation following transduction, flow cytometry was performed using an antibody against the TWIST transcription factor, cells were gated (R1) to exclude debris and cell aggregates, and TWIST-positive cells were subsequently identified within the R1 population (R2). Bar graphs represent the percentage of TWIST-positive cells (R2 gated events %) in (**A**) A549 and (**B**) H1975 cells. To assess the impact of increased Twist presence and its downstream effects, qPCR analysis was conducted to examine the abundance of various epithelial and mesenchymal markers in the A549 cells (**C**,**D**) and H1975 (**E**,**F**) lung cancer cell lines. We compared gene profiles among non-transduced cells, cells transduced with GFP, and cells transduced with Twist. GAPDH and RPL27 were used as housekeeping genes. Relative expression ratios (rERs) were calculated using Schefe’s GED formula [20]. Values are reported as fold changes in relative expression. Data distribution was evaluated using the Shapiro–Wilk normality test, and the data met the assumption of normality. Statistical analysis for group comparisons was performed using one-way ANOVA, with significance set at *p* < 0.05. Results represent three independent biological replicates, each with corresponding technical replicates. “ns” indicates no statistically significant difference, *p* < 0.05 (*), *p* < 0.01 (**), *p* < 0.001 (***) and *p* < 0.0001 (****).

After determining the optimal multiplicity of infection (MOI) for both NSCLC cell lines (Supplementary Figure S1), cells were transduced with N-TWIST Lentiviral Activation Particles and successfully selected using antibiotic resistance (Supplementary Figure S2). TWIST overexpression was evaluated by Western blot (Figure 1A,B), while the expression of epithelial and mesenchymal markers was assessed by qPCR in A549 and NCI-H1975 cells (Figure 1C–F). 

### 3.2. A549 Cells Acquired a Hybrid Epithelial/Mesenchymal Phenotype Following TWIST Overexpression

As shown in Figure 1A, A549 cells transduced with TWIST (A549-Twist) exhibited a significant increase in TWIST protein expression, with approximately 20% of cells testing positive for this transcription factor, whereas fewer than 10% of A549-Control and A549-GFP cells were TWIST-positive. Consistent with these findings, qPCR analysis revealed significant upregulation of the mesenchymal markers TWIST, N-cadherin, and ZEB1 in A549-Twist cells compared with both control groups (Figure 1C). Although Vimentin and Fibronectin were not significantly upregulated, both genes showed a trend toward increased expression.

Interestingly, A549-Twist cells also retained the expression of several epithelial markers. E-cadherin, cytokeratin, and occludin were significantly upregulated, whereas claudin expression was reduced compared with A549-Control and A549-GFP cells (Figure 1D). Collectively, these results indicate that TWIST overexpression in A549 cells induced the simultaneous expression of epithelial and mesenchymal markers, consistent with the acquisition of a hybrid epithelial/mesenchymal (E/M) phenotype.

In contrast, NCI-H1975 cells showed no detectable basal TWIST expression at either the protein (Figure 1B) or mRNA level. Following lentiviral transduction, TWIST overexpression was not achieved (Figure 1B), and no induction of downstream mesenchymal markers was observed. N-cadherin expression was undetectable under all experimental conditions, while Vimentin, Fibronectin, and ZEB1 showed no significant differences among groups (Figure 1E). Vimentin expression was significantly reduced in both H1975-GFP and H1975-Twist cells compared with H1975-Control.

Similarly, epithelial marker expression in H1975 cells showed no pattern consistent with EMT (Figure 1F). E-cadherin expression decreased in H1975-GFP cells but remained unchanged in H1975-Twist cells relative to H1975-Control. Cytokeratin expression increased in H1975-Twist cells, whereas occludin expression decreased in H1975-GFP cells. Claudin expression was reduced in both H1975-GFP and H1975-Twist cells.

Overall, TWIST overexpression successfully induced EMT-associated molecular changes only in A549 cells, whereas NCI-H1975 cells failed to activate a TWIST-dependent transcriptional program under the experimental conditions evaluated.

### 3.3. TWIST Overexpression Promotes Enrichment of CD133^+^ CSCs in A549 Cells

**Figure 2:**
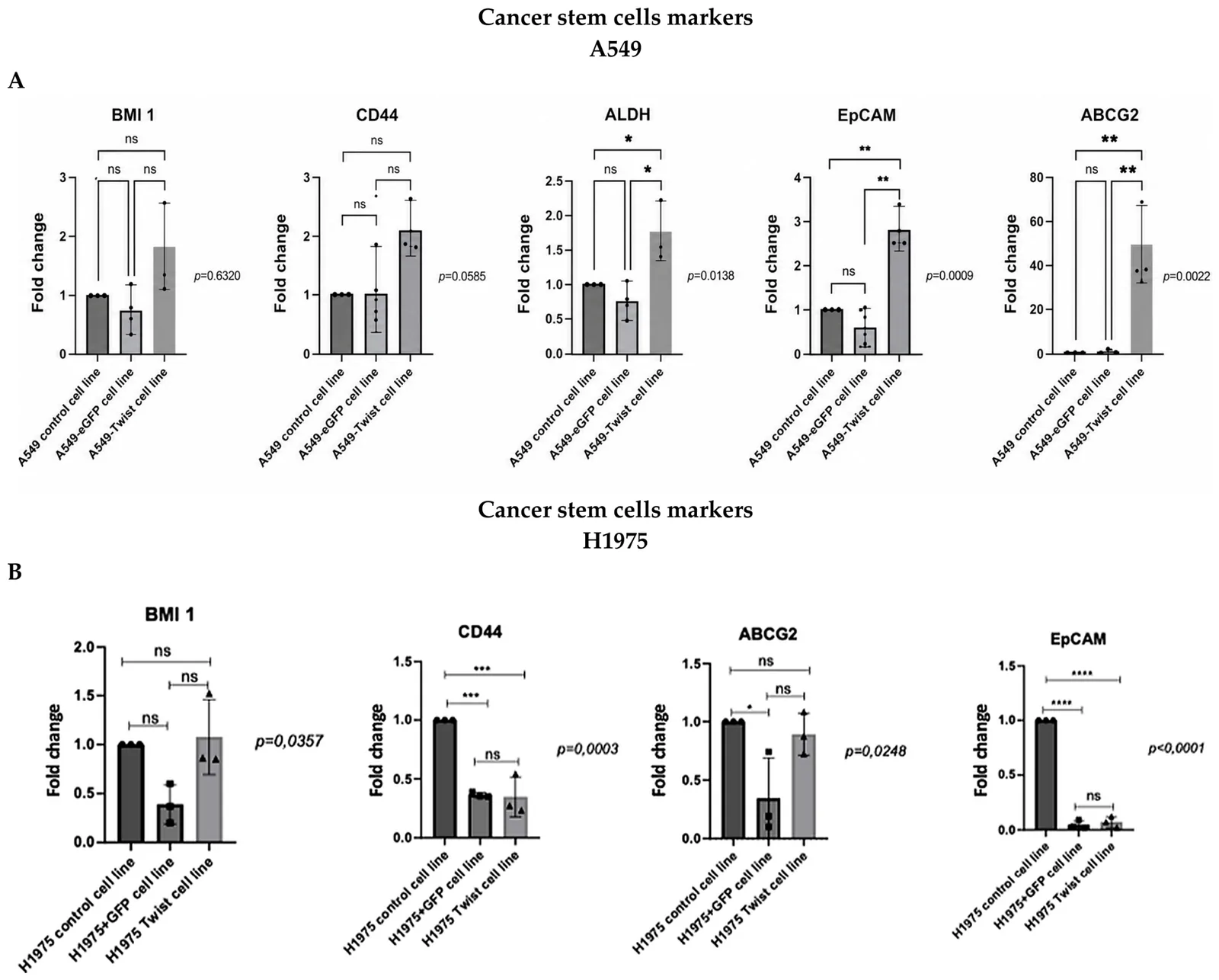
Identification of CSC markers evaluated in transduced lung cancer cell lines. qPCR was performed to assess the presence of various CSC markers in the lung cancer cell lines A549 (**A**) and H1975 (**B**). The results were compared between GFP-transduced and TWIST-induced cells. GAPDH and RPL27 were used as reference genes for normalization. Relative expression ratios (rERs) were calculated using the GED method described by Schefe et al. [20]. Data are presented as fold changes in relative expression. The data are presented as the fold. Data distribution was evaluated using the Shapiro–Wilk normality test, and the data met the assumption of normality. Statistical comparisons among groups were carried out using one-way ANOVA, with significance defined as *p* < 0.05. All findings are based on three independent biological replicates, each analyzed in technical duplicate or triplicate. In the graph, “ns” indicates no statistically significant difference, asterisks indicate levels of significance as follows: *p* < 0.05 (*), *p* < 0.01 (**), *p* < 0.001 (***) and *p* < 0.0001 (****).

To further characterize the phenotypic changes induced by TWIST overexpression, CSC-associated markers were evaluated in both cell lines (Figure 2). As shown in Figure 2A, A549-Twist cells exhibited significantly higher expression of ALDH, ABCG2, and EpCAM compared with A549-Control and A549-GFP cells, indicating the acquisition of CSC-associated molecular features. In contrast, H1975-Twist cells did not show increased expression of CSC markers relative to the corresponding control groups (Figure 2B). ALDH expression was undetectable under all experimental conditions, whereas EpCAM expression was significantly reduced in both H1975-GFP and H1975-Twist cells.

**Figure 3:**
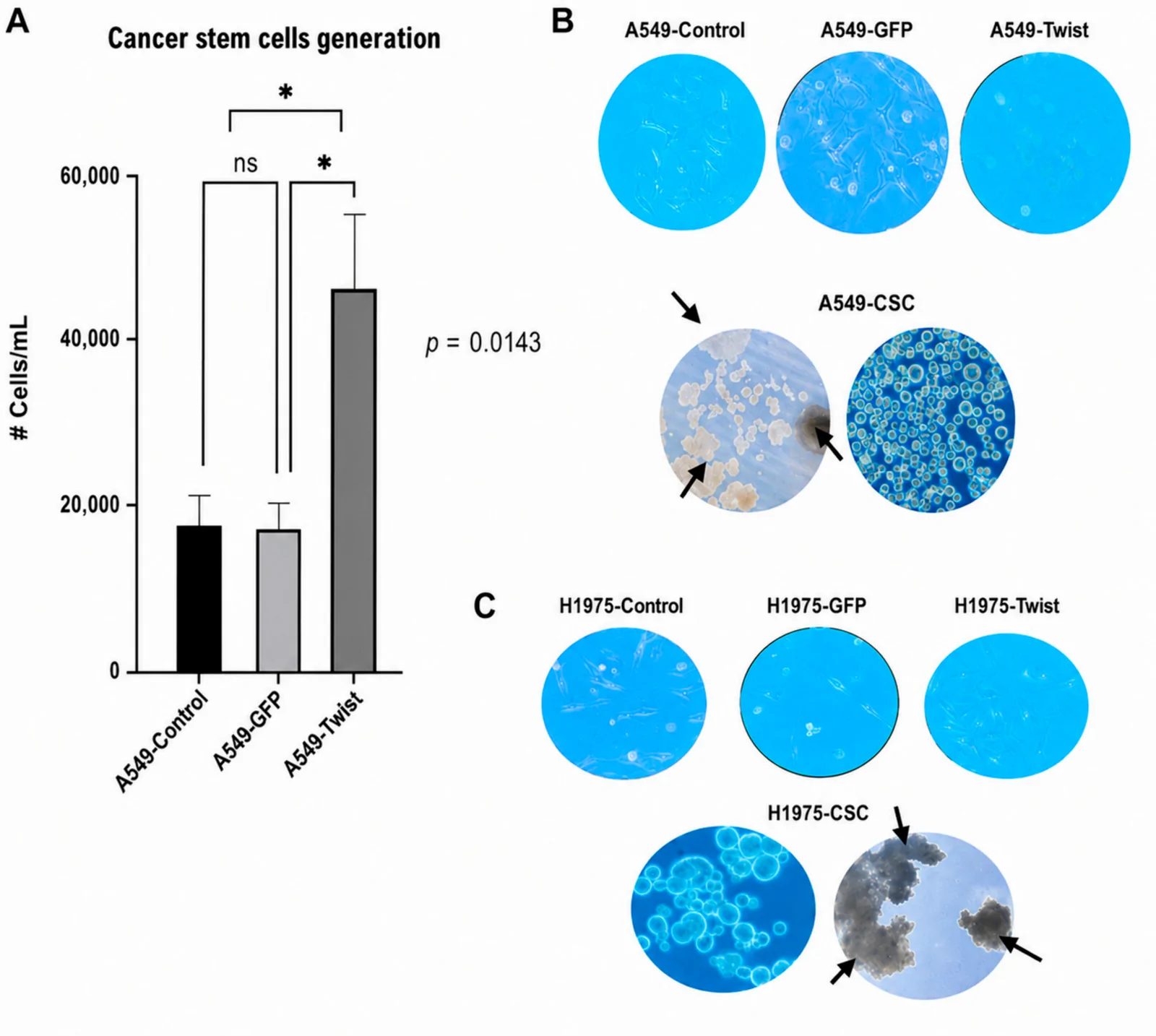
Enrichment and morphology of CD133^+^ CSCs from A549 and H1975 cell lines. (**A**) Quantification of CD133^+^ cells isolated from A549-Control, A549-GFP, and A549-Twist populations after magnetic separation (# Cells/mL = Number of cells per milliliter). Representative bright-field microscopy images of A549 (**B**) and NCI-H1975 (**C**) parental (Control), GFP-transduced (GFP), and TWIST-transduced (Twist) cells cultured under adherent conditions. CD133^+^ CSC-enriched populations were generated by cell sorting and cultured under serum-free, ultra-low attachment conditions supplemented with stem cell growth factors to promote spheroid formation. Under these conditions, CSC-enriched cells formed multicellular spheroids of variable size and compactness. The dense areas observed in some CSC images, indicated by arrows, correspond to compact three-dimensional cellular aggregates formed during spheroid growth. Images are representative of three independent experiments. Data are presented as mean ± SD from three independent biological replicates. Data distribution was evaluated using the Shapiro–Wilk normality test, and the data met the assumption of normality. Statistical analysis was performed using one-way ANOVA followed by Tukey’s post hoc test; “ns” indicates no statistically significant difference and (*) *p* < 0.05 indicates a significant increase in the number of CD133^+^ cells in the A549-Twist population compared to both A549-Control and A549-GFP groups. No significant difference was observed between A549-Control and A549-GFP.

To isolate the CSC-enriched population, cells from each experimental condition were subjected to magnetic separation using an anti-CD133 antibody. A549-Twist cells yielded a significantly higher number of CD133^+^ cells than A549-Control and A549-GFP cells (*p* < 0.0001) (Figure 3A). In contrast, no significant differences in the number of CD133^+^ cells were observed among the H1975 groups.

CD133^+^ cells isolated from both A549-Twist and H1975-Twist populations were subsequently cultured under stem cell-enrichment conditions in ultra-low attachment plates. Bright-field microscopy showed that CD133^+^ cells formed compact spheroids with the characteristic rounded morphology of CSC-enriched cultures in both cell lines (Figure 3B,C), confirming successful enrichment of the CD133^+^ population for subsequent analyses.

We also assessed other characteristics across phenotypes to determine whether additional aspects, such as proliferation and adhesion loss, could vary under different conditions. Regarding proliferation, we observed that the transduction process caused changes in the proliferation rate: A549-GFP cells exhibited increased proliferation compared to A549-Control and A549-Twist cells, while both H1975-GFP and H1975-Twist cells showed higher proliferation than H1975-Control (Supplementary Figure S3). In terms of adhesion, no differences were observed between conditions in either cancer cell line. 

Overall, these results indicate that the success transduction in A549-Twist cell population exhibited gene expression patterns consistent with CSC characteristics, in contrast to H1975-Twist, as expected. However, after culturing under CSC-promoting conditions with the appropriate medium and factors, we were able to obtain CD133^+^ CSC-enriched cells in both cell lines.

### 3.4. 13 microRNAs Were Found Downregulated in CSC Derived from A549 and H1975

Small RNA sequencing was performed on RNA isolated from the four experimental conditions generated for each cell line (Control, GFP, TWIST, and CSC-enriched cells). Differential expression analysis was carried out using DESeq2 by comparing GFP vs. Control, TWIST vs. GFP, and CSC vs. TWIST. Sequencing data have been deposited in the NCBI BioProject database under accession number PRJNA1404308.

**Figure 4:**
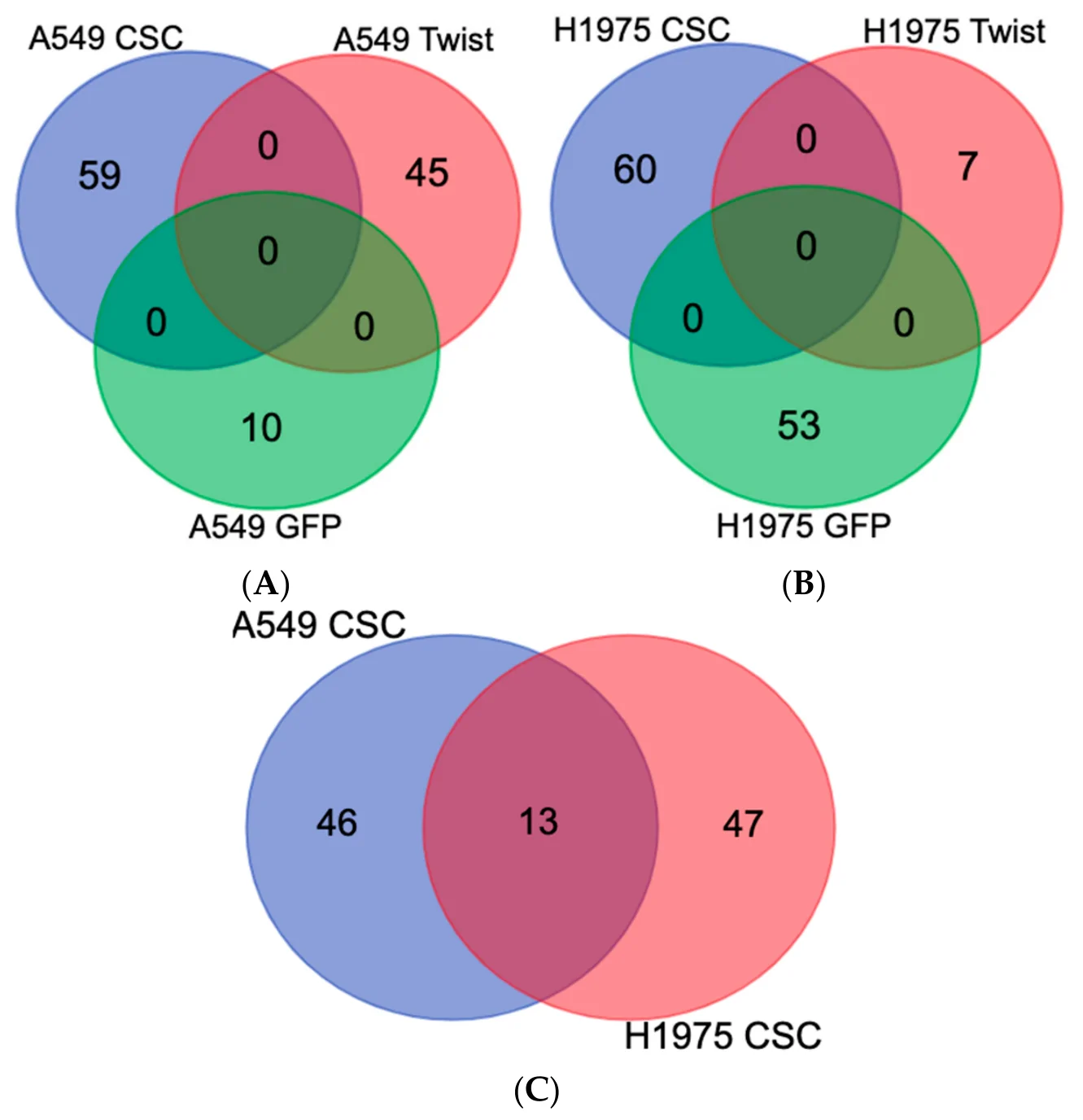
Venn diagrams showing downregulated miRNAs across different conditions in each cell line. (**A**,**B**) The diagrams were constructed using the lists of differentially downregulated miRNAs identified in each comparison group to determine shared and unique miRNAs among conditions. (**C**) The diagram was generated using the lists of miRNAs uniquely downregulated in A549-CSC and H1975-CSC to identify both shared and cell line-specific miRNAs associated with the CSC phenotype. These analyses allowed the exclusion of non-specific miRNAs and facilitated the selection of candidate miRNAs potentially involved in phenotypic transitions. Only miRNAs with an adjusted *p*-value < 0.05, a |log fold change| > 1.5 and Base Mean > 50 were considered.

To identify miRNAs specifically associated with the CSC phenotype while excluding those affected by lentiviral transduction or GFP expression, a sequential filtering strategy based on Venn diagram analysis was applied (Figure 4A,B). Only miRNAs with a log_2_ fold change >1.5, adjusted *p* < 0.05, and baseMean > 50 were included. 

Differential expression analysis identified 59 and 60 uniquely downregulated miRNAs in A549-CSC and H1975-CSC cells, respectively (Figure 4A,B; Supplementary Tables S4 and S7). In contrast, only seven miRNAs were uniquely downregulated in H1975-TWIST cells relative to H1975-GFP cells, whereas 45 miRNAs were uniquely downregulated in A549-TWIST cells compared with A549-GFP cells (Figure 4A; Supplementary Table S6).

No overlap in differentially expressed miRNAs was observed among the GFP, TWIST, and CSC comparisons within either cell line, indicating that the CSC-associated miRNA signature was distinct from changes associated with GFP transduction or TWIST overexpression (Figure 4A,B).

Next, we identified the downregulated miRNAs shared by CD133^+^ CSC-enriched cells from both NSCLC cell lines (Figure 4C). A total of 13 miRNAs were consistently downregulated in both A549-CSC and H1975-CSC populations. Based on the selection criteria (adjusted *p*-value, |log_2_FC|, and baseMean > 50 in both cell lines), hsa-miR-424-3p, hsa-miR-455-3p, hsa-miR-130b-5p, hsa-let-7b-5p, and hsa-miR-15b-5p were identified as the most robust candidates (Table 1). Among these, hsa-miR-424-3p showed the greatest reduction in A549-CSCs (log_2_FC = −3.81), whereas hsa-miR-455-3p exhibited the strongest downregulation in H1975-CSCs (log_2_FC = −3.48). In contrast, hsa-let-7b-5p displayed more moderate fold changes but showed the highest expression levels in both cell lines (baseMean = 21).

### 3.5. Functional Enrichment Analysis of the Common Downregulated CSC-Associated miRNA Signature

To investigate the biological significance of the common downregulated miRNA signature, target genes were filtered using a network degree cutoff of ≥5 and subjected to Kyoto Encyclopedia of Genes and Genomes (KEGG) pathway and Gene Ontology (GO) Biological Process (BP) enrichment analyses.

**Figure 5:**
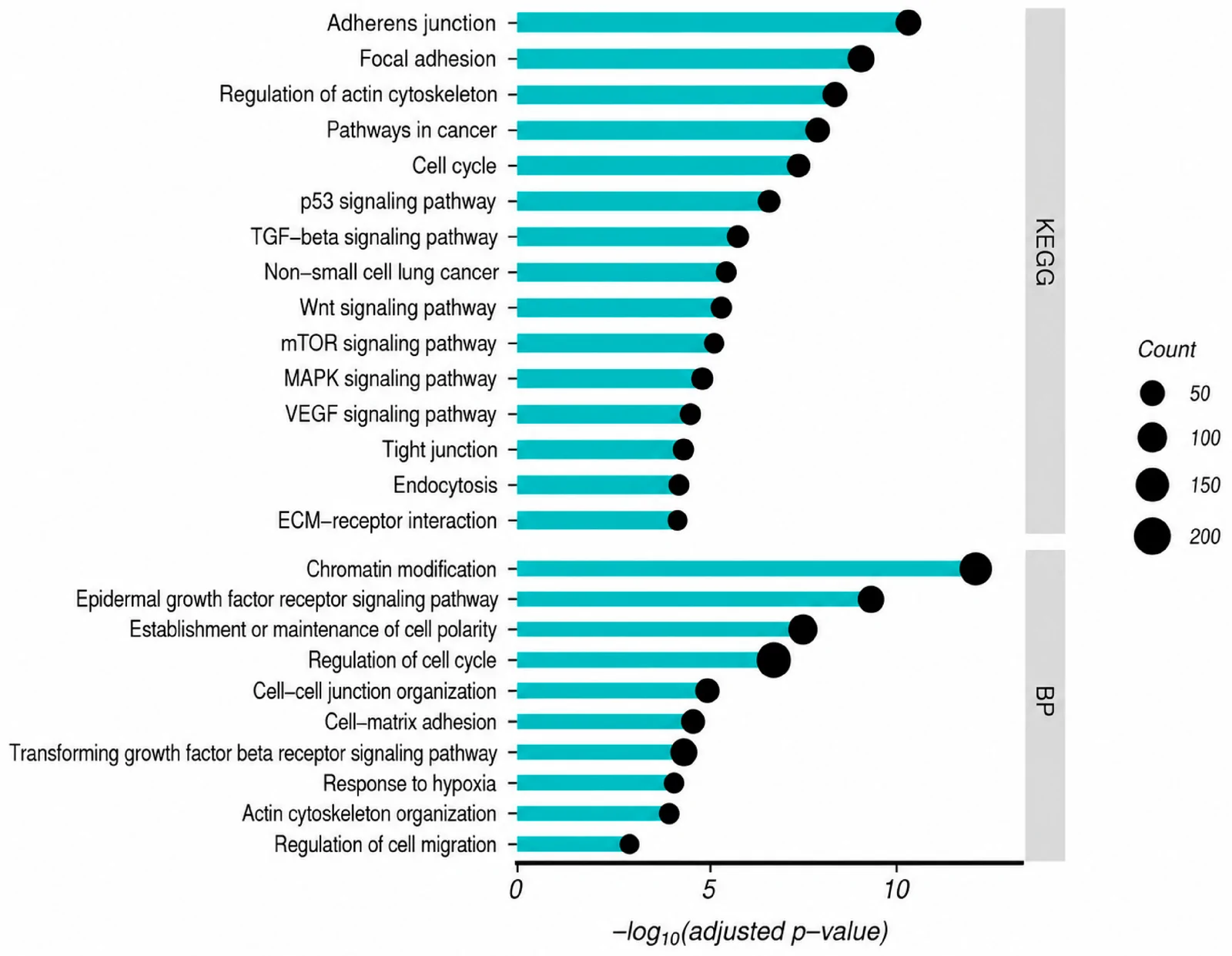
Functional enrichment analysis of pathways and biological processes associated with the target genes of miRNAs downregulated in CSCs from both cell lines. The filtered target gene network (degree cutoff ≥ 5) was subjected to KEGG pathway and GO Biological Process (BP) enrichment analyses. The *x*-axis represents the enrichment significance as −log10 (adjusted *p*-value), where higher values indicate greater statistical significance. The size of each dot corresponds to the number of genes associated with each enriched pathway or biological process (gene count). Representative pathways and biological processes related to epithelial–mesenchymal transition (EMT), cancer stem cell (CSC) was considered. Visual representation generated with SRPLOT, https://www.bioinformatics.com.cn/en (accessed on 26 June 2026).

KEGG pathway analysis revealed significant enrichment of signaling pathways associated with tumor progression, epithelial plasticity, and CSC biology. The most significantly enriched pathways included the Wnt signaling pathway, Cell cycle, TGF-β signaling pathway, Focal adhesion, Regulation of actin cytoskeleton, Adherens junction, MAPK signaling pathway, and ErbB signaling pathway (Figure 5).

### 3.6. miRNA–Target Interaction Network Analysis of the Common Downregulated CSC-Associated miRNA Signature

**Figure 6:**
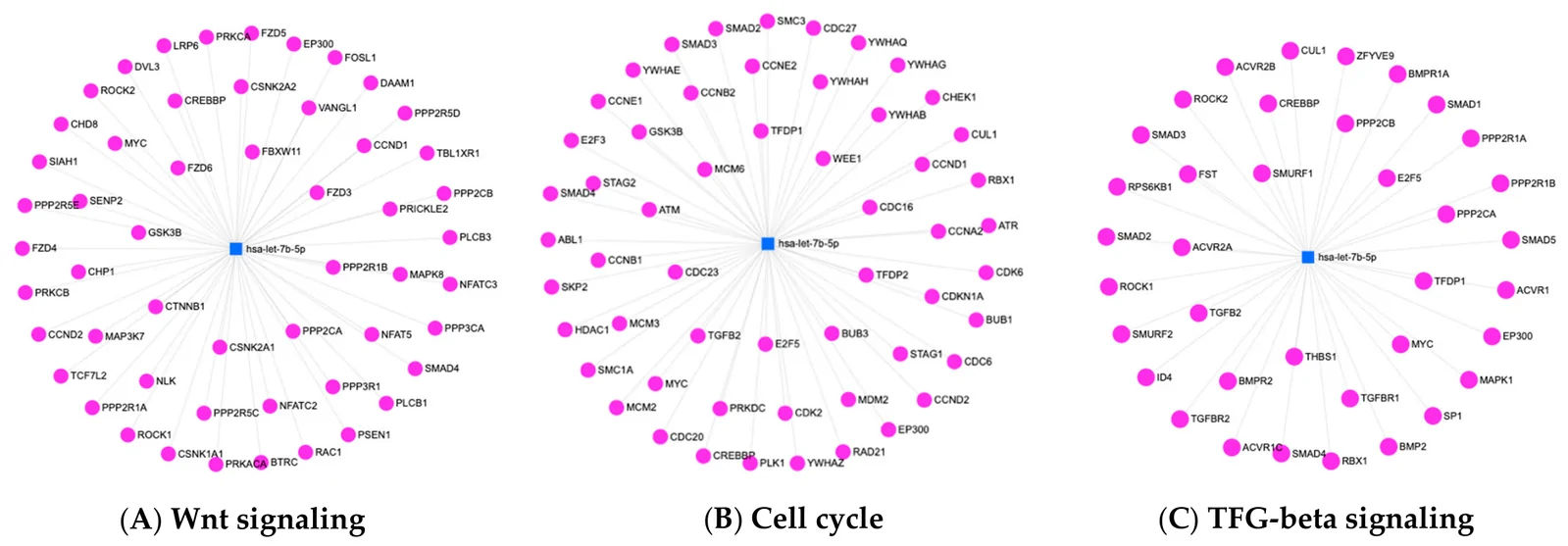
hsa-let-7b-5p interaction networks in representative EMT- and CSC-associated signaling pathways. Interaction networks were constructed from the common downregulated miRNAs in CSCs derived from A549 and H1975 cells. Three representative pathways identified by KEGG enrichment analysis were selected: (**A**) Wnt signaling pathway, (**B**) Cell cycle, and (**C**) TGF-β signaling pathway. The blue square represents hsa-let-7b-5p, while the pink circles represent its predicted target genes within each pathway. Edges indicate predicted miRNA–target gene interactions retrieved through miRNet using the predefined filtering parameters described in the Materials and Methods.

To elucidate the regulatory mechanisms associated with the common downregulated CSC-associated miRNA signature, miRNA–target interaction networks were constructed using miRNet for the three most representative pathways identified by KEGG enrichment analysis: Wnt signaling pathway, Cell cycle, and TGF-β signaling pathway (Figure 6).

Notably, hsa-let-7b-5p emerged as the predominant regulatory miRNA across all three interaction networks, displaying predicted interactions with multiple target genes within each pathway (Figure 6). In the Wnt signaling pathway, hsa-let-7b-5p targeted genes involved in ligand–receptor interactions, intracellular signaling, and transcriptional regulation, including WNT11, FZD3, FZD5, and FZD6 (Figure 6A). Within the Cell cycle network, hsa-let-7b-5p was predicted to regulate key cell cycle components, including ATM, CHEK1, WEE1, and RB1 (Figure 6B). Similarly, in the TGF-β signaling pathway, hsa-let-7b-5p was associated with genes encoding TGF-β receptors (TGFBR1 and TGFBR2), SMAD family members, and the transcription factor MYC (Figure 6C).

Together, these interaction networks identify hsa-let-7b-5p as the principal regulatory miRNA shared across multiple signaling pathways associated with epithelial plasticity, cell cycle regulation, and CSC biology.

### 3.7. 13 microRNAs Were Found Upregulated in CSC Derived from A549 and H1975

**Figure 7:**
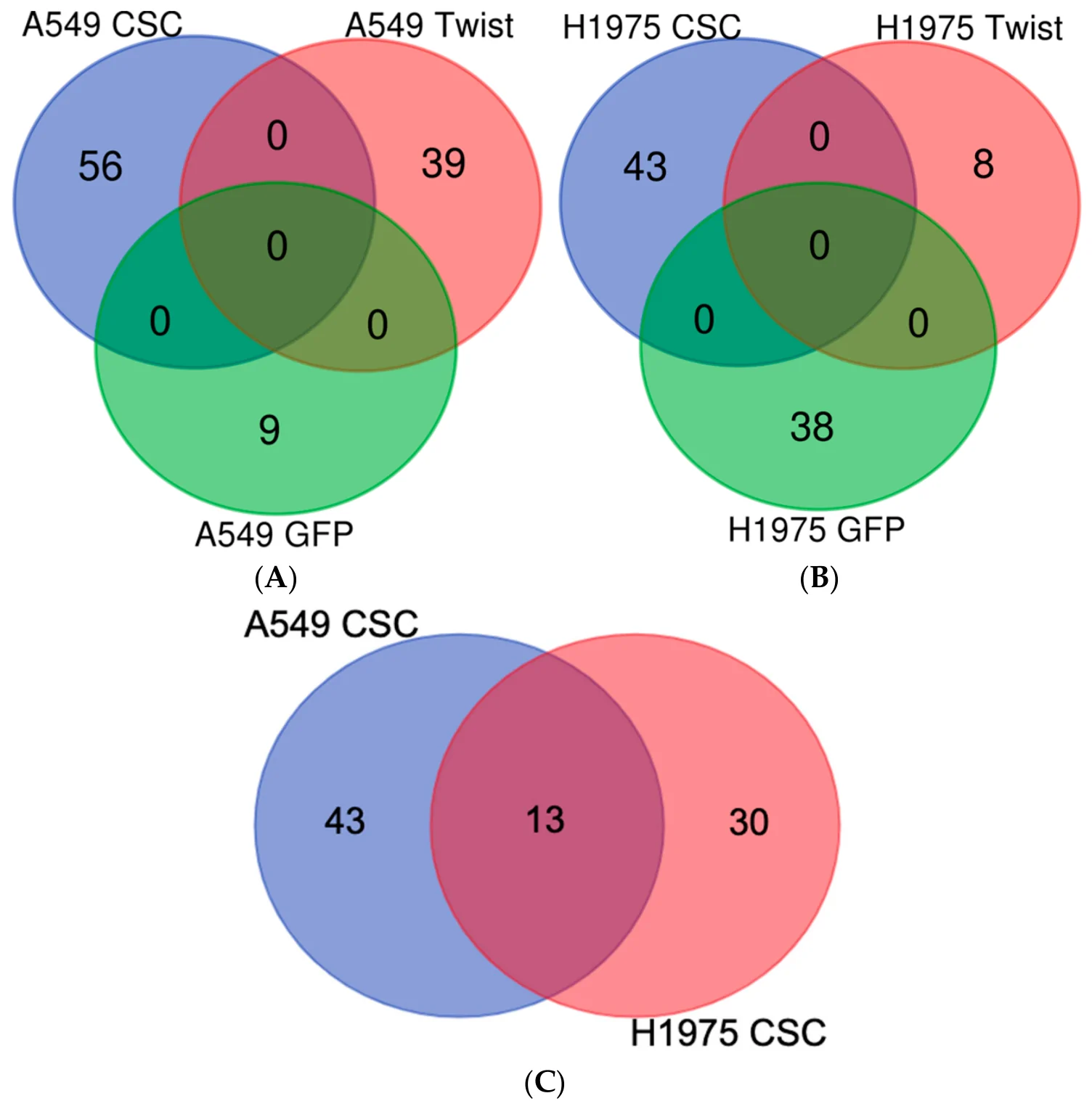
Venn diagram showing the unique upregulated miRNAs across different conditions for each cell line. (**A**,**B**) The diagram was generated based on the lists of miRNAs significantly upregulated in each comparison group, aiming to identify both common and condition-specific miRNAs. (**C**) The diagram was created using the lists of miRNAs that showed exclusive upregulation in A549-CSC and H1975-CSC, with the objective of identifying miRNAs that are either shared between or specific to each cell line in relation to the CSC phenotype. This approach helped eliminate non-specific candidates and supported the selection of miRNAs potentially involved in the observed phenotypic changes. Only miRNAs with an adjusted *p*-value < 0.05, a |log fold change| > 1.5 and Base Mean > 50 were considered.

As shown in Figure 7A,B and Supplementary Tables S4 and S7, GFP transduction resulted in the differential expression of several miRNAs, particularly in H1975 cells. In A549-TWIST cells, 39 miRNAs were uniquely upregulated compared with A549-GFP cells (Figure 7A; Supplementary Table S3), whereas only eight miRNAs were uniquely upregulated in H1975-TWIST cells relative to H1975-GFP cells (Figure 7B; Supplementary Table S6).

Analysis of the CSC populations identified 56 and 43 uniquely upregulated miRNAs in A549-CSC and H1975-CSC cells, respectively (Figure 7A,B; Supplementary Tables S4 and S7). No overlap in differentially expressed miRNAs was observed among the GFP, TWIST, and CSC comparisons within either cell line.

Subsequent intersection of the CSC-associated miRNA signatures identified 13 miRNAs consistently upregulated in CD133^+^ CSC-enriched cells derived from both NSCLC cell lines (Figure 7C). Based on the selection criteria (adjusted *p*-value, |log_2_FC|, and baseMean > 50 in both cell lines), hsa-miR-1246, hsa-miR-582-3p, hsa-miR-101-3p, hsa-miR-374a-5p, and hsa-let-7a-3p were identified as the most robust candidates (Table 2).

Among these, hsa-miR-1246 exhibited the greatest increase in H1975-CSCs (log_2_FC = 7.08), whereas hsa-miR-582-3p and hsa-miR-374a-5p showed the largest fold changes in A549-CSCs (log_2_FC = 5.90 and 5.57, respectively). In contrast, hsa-miR-101-3p displayed more moderate fold changes but exhibited relatively high expression levels in both cell lines (baseMean = 1155.34 in A549-CSCs and 2123.71 in H1975-CSCs). Likewise, hsa-let-7a-3p showed highly consistent fold changes and adjusted *p*-values across both CSC models (Table 2).

These commonly upregulated miRNAs were subsequently selected for functional enrichment and miRNA–target interaction network analyses.

### 3.8. Functional Enrichment Analysis of the Common Upregulated CSC-Associated miRNA Signature

**Figure 8:**
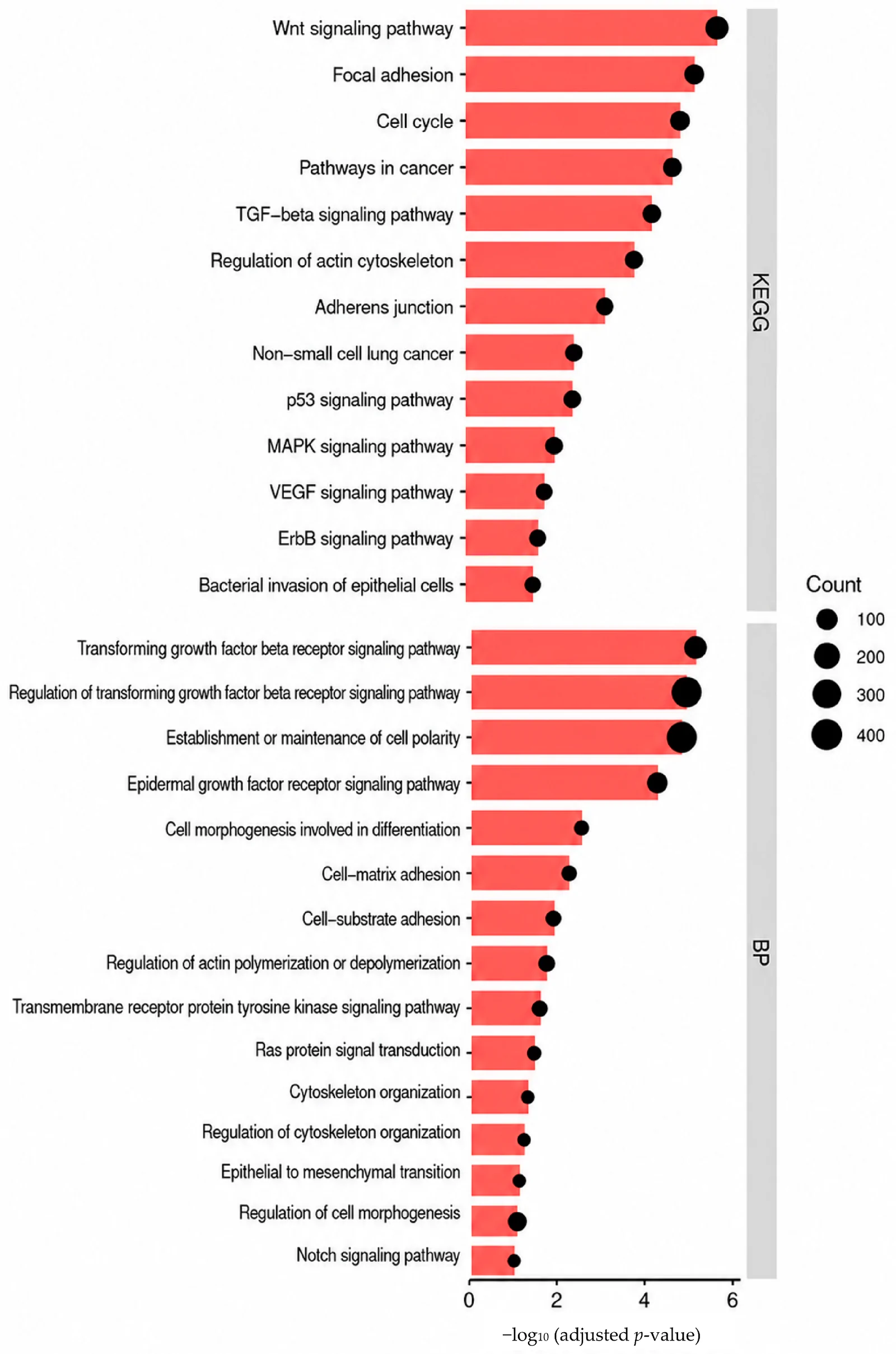
Functional enrichment analysis of pathways and biological processes associated with the target genes of miRNAs upregulated in CSCs from both cell lines. The filtered target gene network (degree cutoff ≥ 5) was subjected to Kyoto Encyclopedia of Genes and Genomes (KEGG) pathway and Gene Ontology (GO) Biological Process (BP) enrichment analyses. Only pathways and biological processes associated with epithelial–mesenchymal transition (EMT) and cancer stem cell (CSC) biology are shown. The *x*-axis represents the enrichment significance as −log_10_ (adjusted *p*-value), where larger values indicate higher statistical significance. The size of each dot corresponds to the number of enriched genes (Count) associated with each pathway or biological process. Visual representation generated with SRPLOT, https://www.bioinformatics.com.cn/en (accessed on 26 June 2026).

To investigate the biological functions associated with the target genes of the 13 miRNAs commonly upregulated in CD133^+^ CSC-enriched cells from both NSCLC cell lines, Kyoto Encyclopedia of Genes and Genomes (KEGG) pathway and Gene Ontology (GO) Biological Process enrichment analyses were performed (Figure 8).

KEGG and GO analyses identified significant enrichment of pathways and biological processes associated with epithelial plasticity, cancer stem cell biology, cell proliferation, migration, cell adhesion, cytoskeletal organization, and growth factor-mediated signaling (Figure 8). The most significantly enriched KEGG pathways included Wnt signaling, TGF-β signaling, mTOR signaling, focal adhesion, adherens junction, regulation of the actin cytoskeleton, and MAPK signaling, among others.

### 3.9. miRNA–Target Interaction Network Analysis of the Common Upregulated CSC-Associated miRNA Signature

**Figure 9:**
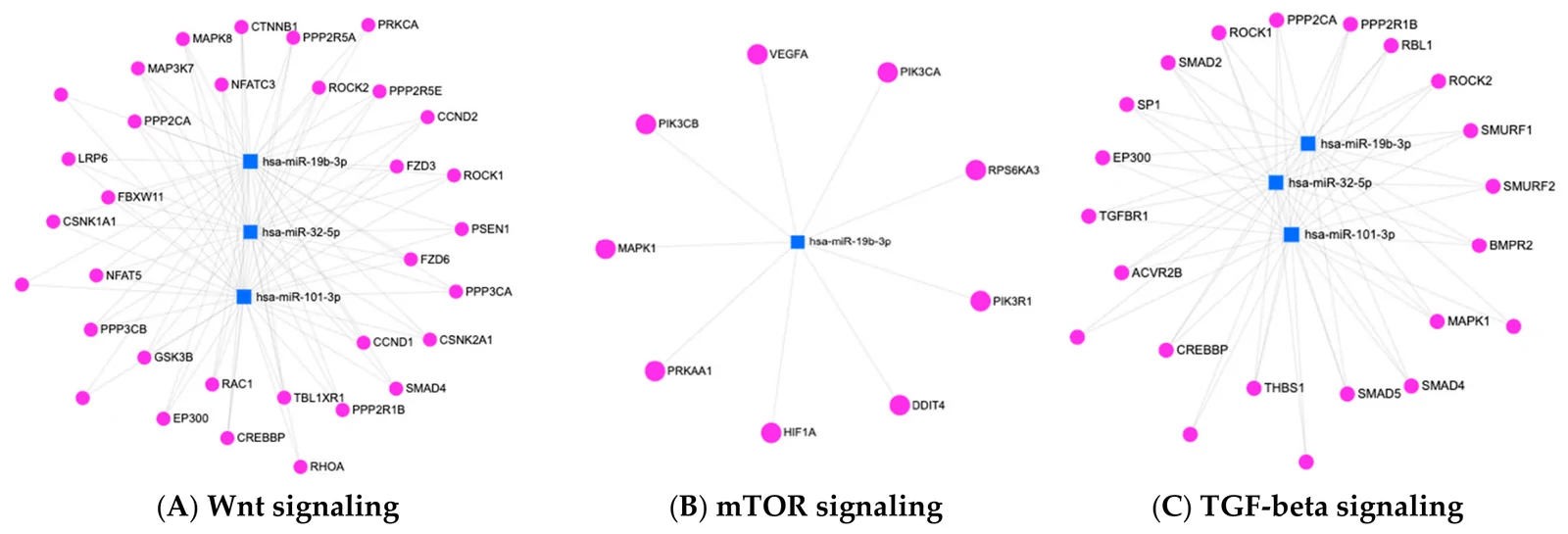
miRNA–target gene interaction networks of the principal signaling pathways associated with EMT and CSC biology. Interaction networks were constructed from the common upregulated miRNAs in CSCs derived from A549 and H1975 cells. Three representative pathways identified by KEGG enrichment analysis were selected: (**A**) Wnt signaling pathway, (**B**) mTOR signaling pathway, and (**C**) TGF-β signaling pathway. Blue squares represent the upregulated miRNAs, pink circles represent target genes, and gray edges indicate validated miRNA–target gene interactions. Only genes associated with EMT- and CSC-related pathways are displayed.

To further characterize the regulatory interactions associated with the common upregulated CSC-associated miRNA signature, miRNA–target interaction networks were constructed using miRNet for three representative pathways identified by KEGG enrichment analysis: Wnt signaling, mTOR signaling, and TGF-β signaling (Figure 9).

Within the Wnt signaling pathway, the common upregulated miRNAs were predicted to target genes involved in ligand–receptor interactions, intracellular signaling, and transcriptional regulation, including LRP6, FZD3, FZD6, CTNNB1, GSK3B, ROCK1, RAC1, RHOA, CSNK1A1, CREBBP, EP300, SMAD4, NFATC3, and CCND1 (Figure 9A). Among the identified miRNAs, hsa-miR-101-3p exhibited the highest degree of connectivity within this network.

The mTOR signaling pathway network included genes associated with growth factor signaling, nutrient sensing, and cell survival, including PIK3CA, PIK3R1, PIK3CB, MAPK1, VEGFA, HIF1A, PRKAA1, DDIT4, and RPS6KA3 (Figure 9B). Within this network, hsa-miR-19b-3p displayed the highest degree of connectivity.

Similarly, in the TGF-β signaling pathway, the common upregulated miRNAs were associated with genes encoding receptors, intracellular mediators, and transcriptional regulators, including TGFBR1, ACVR2B, BMPR2, SMAD2, SMAD3, SMAD4, ROCK1, ROCK2, MAPK1, CREBBP, EP300, SP1, THBS1, SMURF1, and SMURF2 (Figure 9C). As observed in the Wnt signaling network, hsa-miR-101-3p exhibited the highest degree of connectivity.

Together, these interaction networks identify hsa-miR-101-3p and hsa-miR-19b-3p as the principal regulatory miRNAs within the enriched pathways associated with the common upregulated CSC-associated miRNA signature.

### 3.10. 45 microRNAs Were Significantly Downregulated During TWIST-Induced EMT in A549 Cells

Subsequently, the miRNA expression profile associated with TWIST-induced EMT was analyzed in A549 cells (Supplementary Table S3). A total of 45 miRNAs were significantly downregulated following TWIST induction. Among these, several members of the miR-200 family, including hsa-miR-200a-3p, hsa-miR-200a-5p, hsa-miR-200b-3p, and hsa-miR-200b-5p, were consistently downregulated (Table 3).

**Figure 10:**
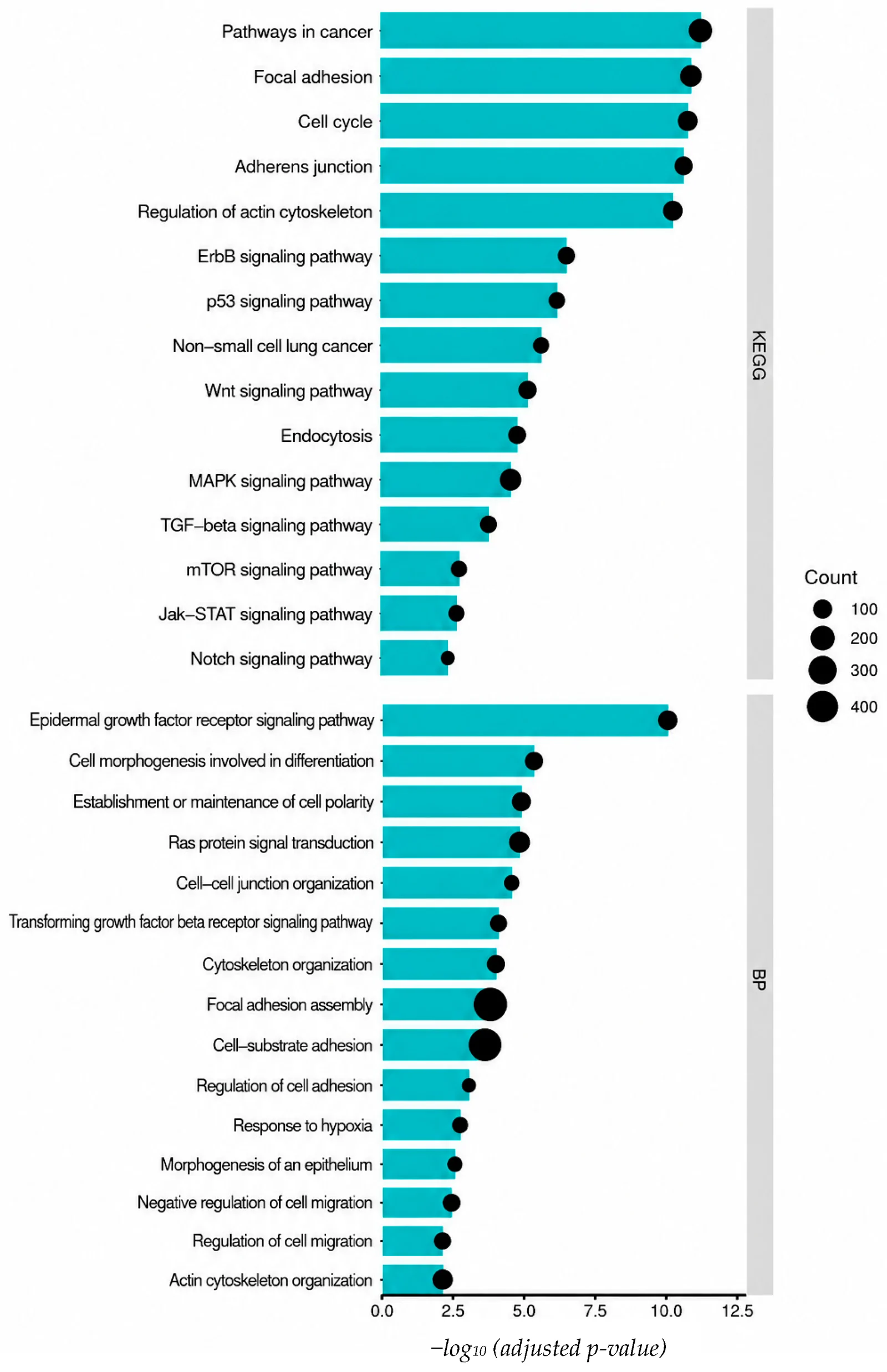
KEGG pathway and Gene Ontology (GO) Biological Process (BP) enrichment analyses of the target genes of downregulated miRNAs in A549-Twist. The analyses were performed using the filtered target gene network generated in miRNet (degree cutoff ≥ 5). Only pathways and biological processes related to epithelial–mesenchymal transition (EMT) were considered. Bars represent the enrichment significance (−log_10_ adjusted *p*-value), and dot size indicates the number of enriched genes (Count). Visual representation generated with SRPLOT, https://www.bioinformatics.com.cn/en (accessed on 26 June 2026).

To functionally characterize the downregulated EMT-associated miRNA signature, predicted target genes were subjected to Kyoto Encyclopedia of Genes and Genomes (KEGG) pathway and Gene Ontology (GO) Biological Process enrichment analyses. KEGG analysis identified significant enrichment of pathways involved in cell proliferation, cell adhesion, cell migration, cytoskeletal organization, and growth factor-mediated signaling. Likewise, GO Biological Process analysis revealed enrichment of processes related to growth factor receptor signaling, extracellular matrix organization, cell morphogenesis, cell adhesion, regulation of cell population proliferation, cytoskeletal organization, and response to hypoxia (Figure 10).

### 3.11. miRNA–Target Interaction Network Analysis of the Downregulated EMT-Associated miRNA Signature

To further characterize the regulatory interactions associated with the downregulated EMT-associated miRNA signature, miRNA–target interaction networks were constructed using miRNet for the TGF-β signaling and Adherens junction pathways, two of the most significantly enriched pathways identified by KEGG analysis (Figure 10).

**Figure 11:**
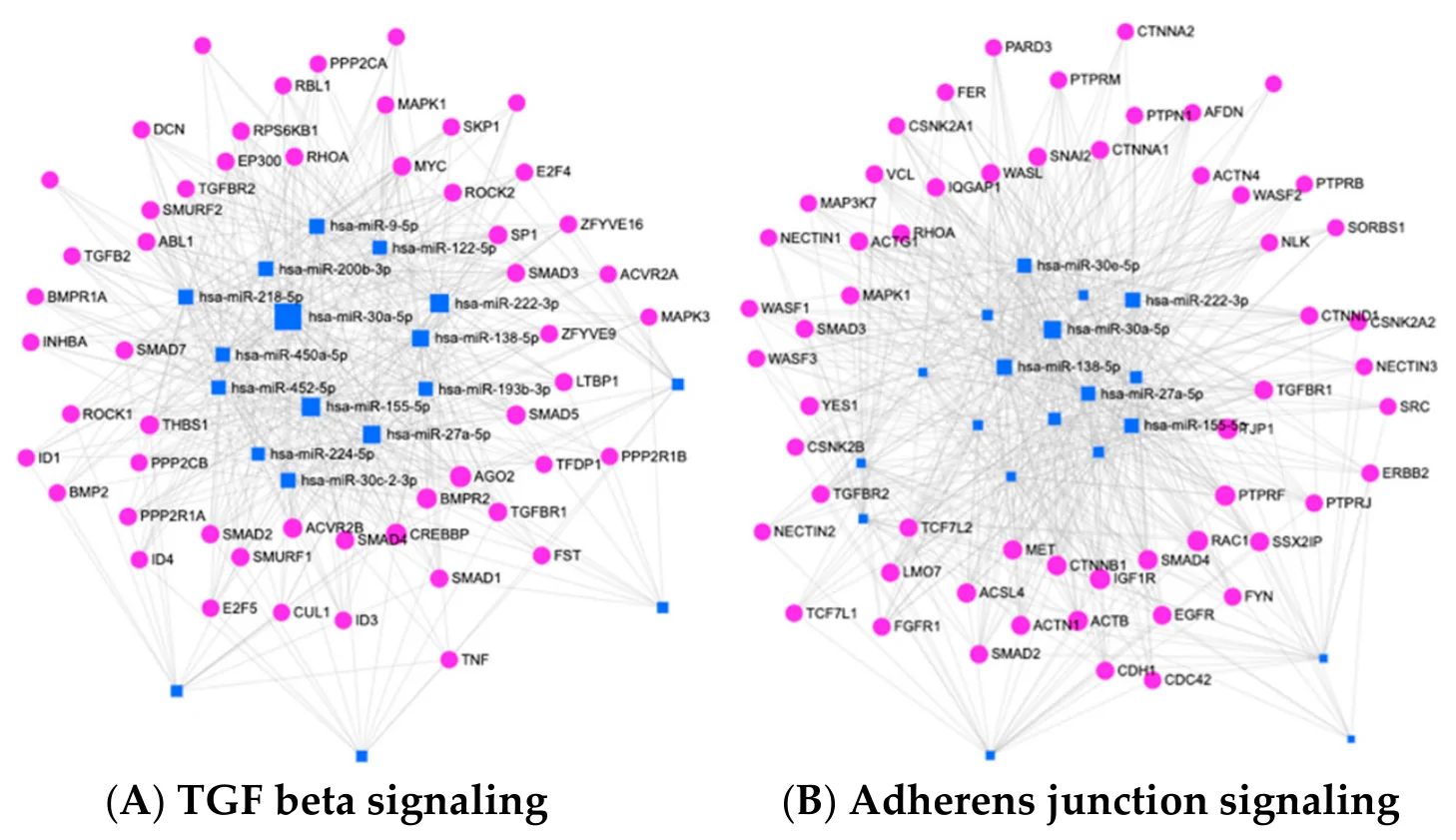
miRNA–target gene interaction networks of representative EMT-related signaling pathways. Target genes of the commonly downregulated miRNAs in A549-Twist were filtered using a degree cutoff ≥ 5. (**A**) TGF-β signaling and (**B**) Adherens junction signaling pathways. Blue squares represent downregulated miRNAs, pink circles represent target genes, and gray edges indicate miRNA–target gene interactions. These pathways were selected as representative networks because they contained the largest number of interactions among the enriched EMT-associated pathways.

Within the TGF-β signaling pathway, the downregulated miRNAs were predicted to target genes encoding ligands, receptors, intracellular mediators, and transcriptional regulators, including TGFB1, TGFB2, TGFBR1, TGFBR2, ACVR2A, ACVR2B, BMPR1A, BMPR2, SMAD family members, ROCK1, ROCK2, CREBBP, EP300, MYC, and ZFYVE16 (Figure 11A). Several target genes were connected to multiple miRNAs within this interaction network.

Similarly, in the Adherens junction pathway, the downregulated miRNAs were associated with genes involved in cell–cell adhesion, cytoskeletal organization, and intracellular signaling, including CTNNA1, CTNNA2, CTNNB1, CDH1, ACTN4, SRC, EGFR, ERBB2, MET, TGFBR1, SMAD2, SMAD4, CDC42, WASF2, PARD3, and NECTIN2 (Figure 11B). Multiple interactions were also identified for CTNNB1, EGFR, MET, TGFBR1, and SMAD4, which exhibited the highest degrees of connectivity within the network.

Together, these interaction networks show that multiple downregulated miRNAs converge on a common set of target genes involved in TGF-β signaling and adherens junction organization, highlighting extensive regulatory interactions within pathways associated with epithelial plasticity.

### 3.12. 39 microRNAs Were Significantly Upregulated During TWIST-Induced EMT in A549 Cells

The miRNA expression profile associated with TWIST-induced EMT was subsequently analyzed in A549 cells (Supplementary Table S3). A total of 39 miRNAs were significantly upregulated following TWIST induction. Among these, hsa-miR-1260a and hsa-miR-1260b exhibited both marked upregulation and high expression levels (baseMean = 10,037.92 and 10,563.17, respectively). Although hsa-miR-181a-5p and hsa-let-7c-5p displayed more moderate fold changes, they were among the most highly expressed miRNAs identified (baseMean = 81,055.53 and 9020.67, respectively) (Supplementary Table S3).

**Figure 12:**
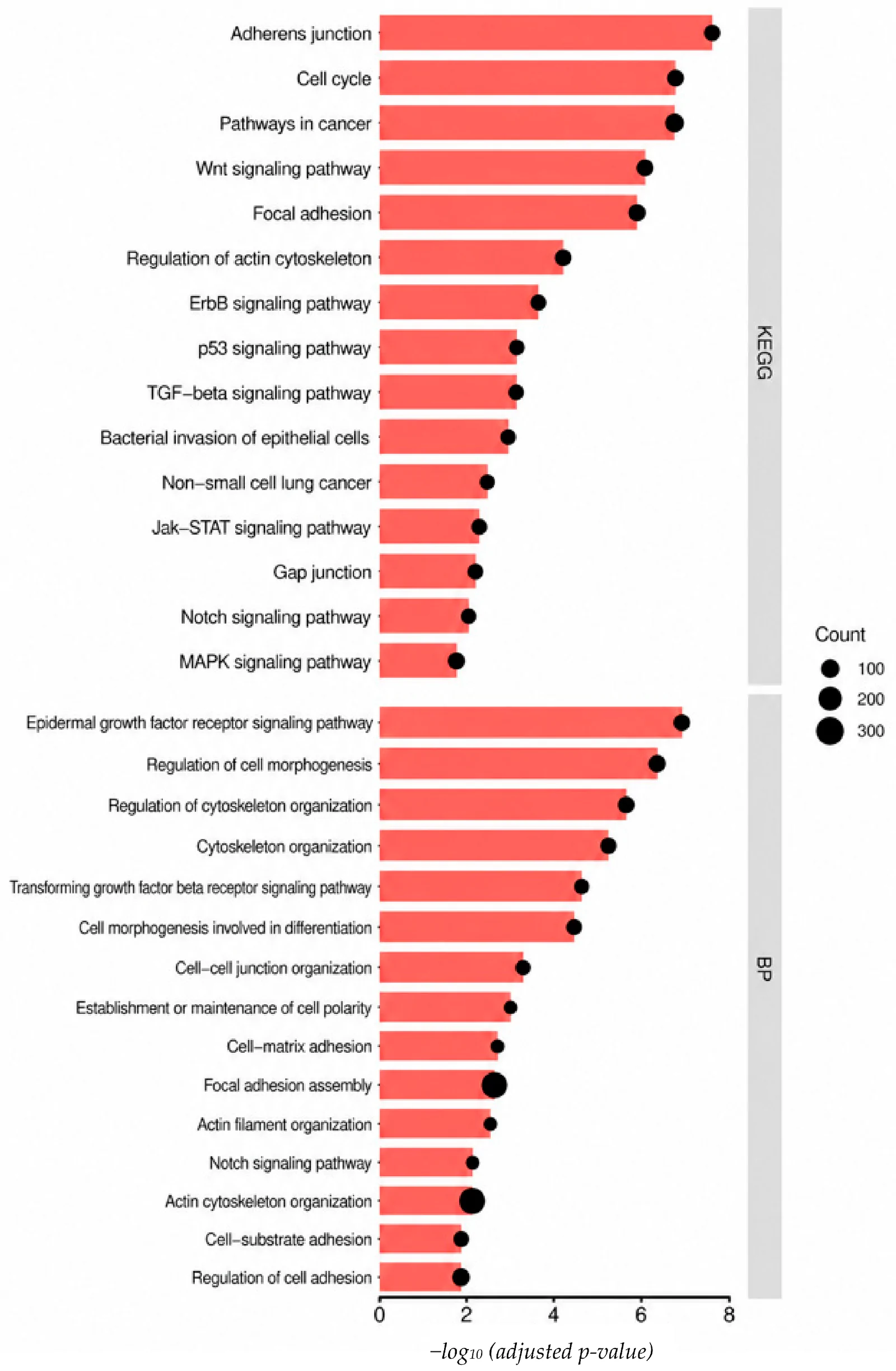
KEGG pathway and Gene Ontology (GO) Biological Process (BP) enrichment analyses of the target genes of upregulated miRNAs in A549-Twist. The analyses were performed using the filtered target gene network generated in miRNet (degree cutoff ≥ 5). Only pathways and biological processes related to epithelial–mesenchymal transition (EMT) were considered. Bars represent the enrichment significance (−log_10_ adjusted *p*-value), and dot size indicates the number of enriched genes (Count). Visual representation generated with SRPLOT, https://www.bioinformatics.com.cn/en (accessed on 26 June 2026).

To functionally characterize the upregulated EMT-associated miRNA signature, predicted target genes were subjected to Kyoto Encyclopedia of Genes and Genomes (KEGG) pathway and Gene Ontology (GO) Biological Process enrichment analyses (Figure 12). KEGG analysis identified significant enrichment of pathways related to Wnt signaling, focal adhesion, and regulation of the actin cytoskeleton. Similarly, GO Biological Process analysis revealed enrichment of processes associated with extracellular matrix organization, cell–cell junction organization, cell–matrix adhesion, and epithelial cell differentiation (Figure 12).

### 3.13. miRNA–Target Interaction Network Analysis of the Upregulated EMT-Associated miRNA Signature

To further characterize the regulatory interactions associated with the upregulated EMT-associated miRNA signature, miRNA–target interaction networks were constructed using miRNet for three representative pathways identified by KEGG enrichment analysis: TGF-β signaling, Focal adhesion, and Regulation of the actin cytoskeleton (Figure 12).

**Figure 13:**
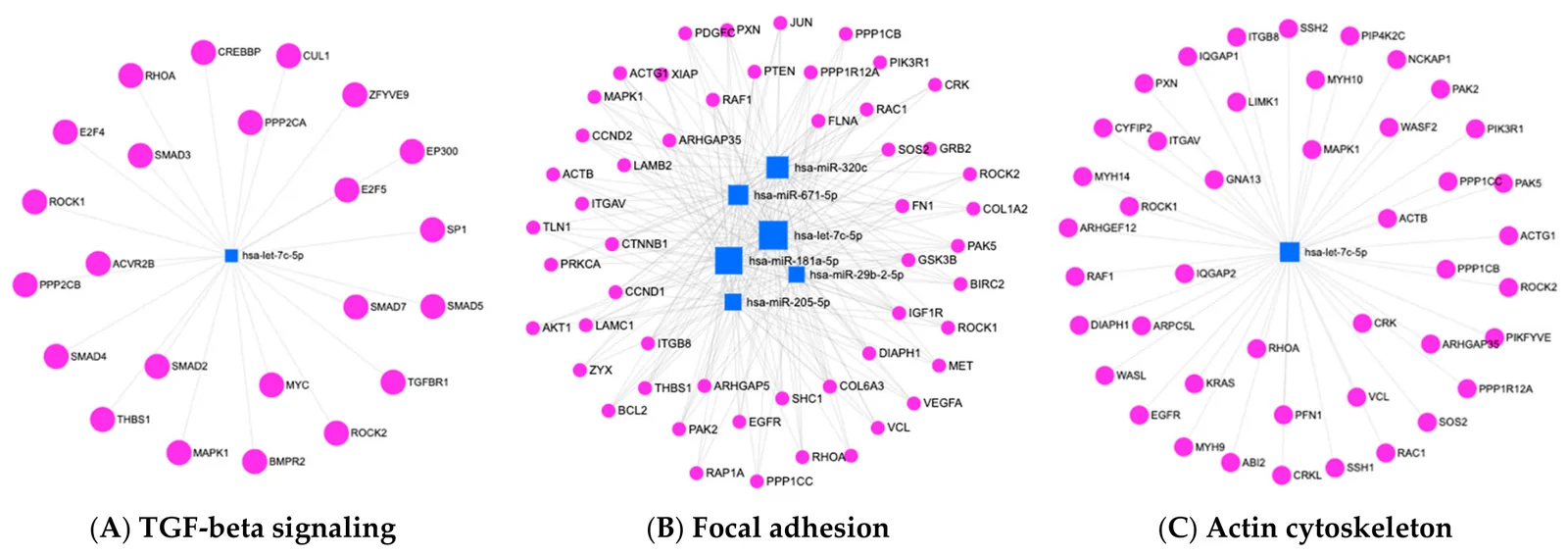
miRNA–target gene interaction networks of representative EMT-related signaling pathways. Target genes of the commonly upregulated miRNAs in A549-Twist were filtered using a degree cutoff ≥ 5. (**A**) TGF-β signaling, (**B**) Focal adhesion signaling pathways and (**C**) Actin cytoskeleton. Blue squares represent downregulated miRNAs, pink circles represent target genes, and gray edges indicate miRNA–target gene interactions. These pathways were selected as representative networks because they contained the largest number of interactions among the enriched EMT-associated pathways.

Within the TGF-β signaling pathway, hsa-let-7c-5p emerged as the predominant miRNA, displaying predicted interactions with genes encoding receptors, intracellular mediators, and transcriptional regulators, including TGFBR1, ACVR2B, BMPR2, SMAD2, SMAD3, SMAD4, SMAD5, ROCK1, ROCK2, CREBBP, EP300, MYC, SP1, and THBS1 (Figure 13A).

In contrast, the Focal adhesion pathway exhibited a more complex interaction network, in which multiple miRNAs were connected to genes involved in integrin signaling, cytoskeletal organization, and growth factor-mediated signaling. Representative target genes included EGFR, MET, ITGA5, ITGB1, PXN, VCL, ACTN1, ACTN4, RAC1, ROCK1, ROCK2, PIK3R1, VEGFA, CCND1, CCND2, and BCL2 (Figure 13B).

Similarly, in the Regulation of the actin cytoskeleton pathway, hsa-let-7c-5p exhibited the highest degree of connectivity, with predicted interactions involving genes associated with actin dynamics and Rho GTPase signaling, including RHOA, RAC1, ROCK1, ROCK2, ACTB, ACTG1, VCL, PXN, PAK2, PAK5, KRAS, EGFR, PIK3R1, WASF2, and MYH9 (Figure 13C).

Together, these interaction networks identify hsa-let-7c-5p as the predominant regulatory miRNA in the TGF-β signaling and Regulation of the actin cytoskeleton pathways, whereas the Focal adhesion pathway displayed a more distributed interaction pattern involving multiple miRNAs.

### 3.14. hsa-let-7a-3p Is Associated with Signaling Pathways Related to CSC Biology

To select a candidate miRNA for functional validation, the commonly upregulated miRNAs identified in CD133^+^ CSC-enriched cells from both NSCLC cell lines were prioritized based on literature reported, adjusted *p*-value, fold change, and consistency of differential expression across both models. Based on these criteria, hsa-let-7a-3p was selected for subsequent analyses (Table 2).

To investigate the biological processes potentially regulated by hsa-let-7a-3p, experimentally validated target genes were retrieved from the TarBase v9 database (Supplementary Table S8). Target genes with microT scores between 0.9 and 1.0 were selected for downstream functional enrichment analyses.

**Figure 14:**
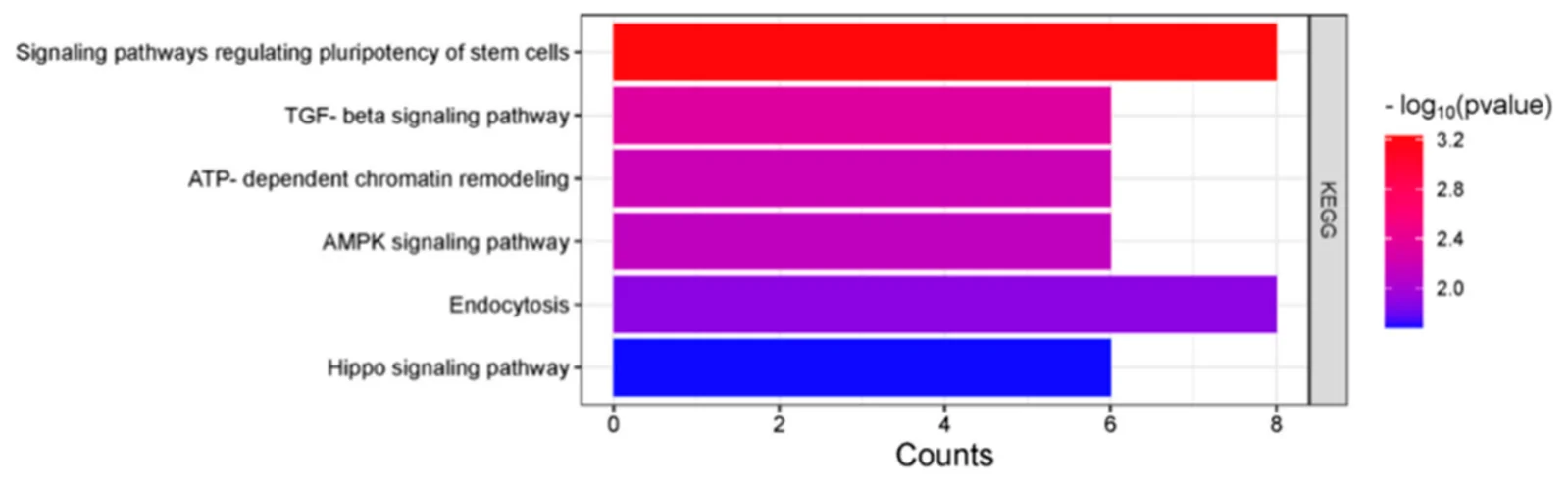
Pathway enrichment analysis. Pathways reported in the enrichment functional analysis from the genes regulated by hsa-let-7a-3p through the DAVID tool were considered. Pathways with a FDR < 0.05 included, particularly those that could be related to the maintenance of cancer stem cells (visual representation generated with SRPLOT, https://www.bioinformatics.com.cn/en (accessed on 10 October 2025). The *x*-axis represents the number of genes involved in each pathway, and the *y*-axis shows the different pathways associated with CSCs. The color of each bar represents the −log10 of the *p*-value, where more significant *p*-values correspond to higher −log10(*p*) values. Thus, red indicates higher statistical significance, while blue indicates lower significance.

KEGG pathway enrichment analysis revealed that the validated target genes of hsa-let-7a-3p were significantly enriched in signaling pathways associated with stem cell pluripotency, TGF-β signaling, Wnt signaling, mTOR signaling, focal adhesion, and regulation of the actin cytoskeleton, among others (Figure 14 and Supplementary Table S9).

To further validate these findings, pathway enrichment analysis was independently performed using DIANA-miRPath v4.0 using the same filtered target gene set (Supplementary Table S8). The enriched pathways were largely consistent with those identified by the KEGG analysis and included the PI3K–AKT signaling pathway, focal adhesion, and other cancer-related pathways (Table 3).

### 3.15. hsa-let-7a-3p Is Overexpressed in CD133^+^ CSC-Enriched Cells Derived from A549 and H1975

To validate the small RNA-seq results, hsa-let-7a-3p expression was evaluated by qPCR in control, GFP, Twist, and CD133^+^ CSC-enriched cells from both NSCLC cell lines.

**Figure 15:**
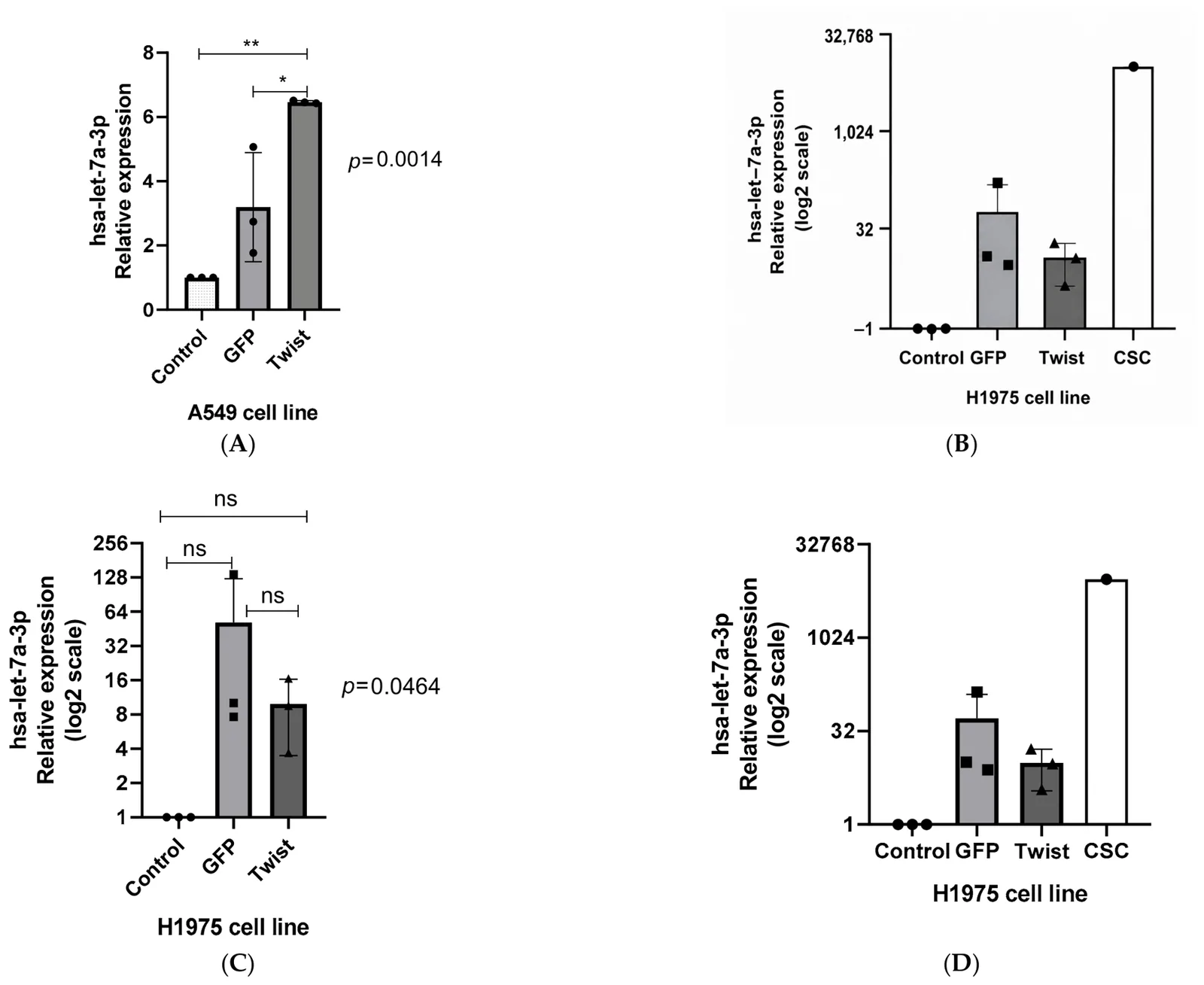
qPCR analysis of hsa-let-7a-3p expression in A549 and H1975 cell lines under different phenotypic conditions. (**A**) Relative expression in A549 control, GFP, and Twist cells. Data are shown as mean ± [SD/SEM] from three biological replicates with technical replicates; “ns” indicates no statistically significant difference, one-way ANOVA. (**B**) Expression in A549-derived CSCs. Results are shown descriptively (two biological replicates), showing the same directionality, and supporting RNA-seq data; no statistical analysis was performed. Axis scaling was adjusted for visualization. (**C**) Relative expression in H1975 control, GFP, and Twist cells. Data represent mean ± [SD/SEM] from three biological replicates with technical replicates; Kruskal–Wallis test was used with significance defined as *p* < 0.05. “ns” indicates no statistically significant difference. (**D**) Expression in H1975-derived CSCs. Data are shown descriptively (one biological replicate), showing a trend toward increased expression consistent with RNA-seq results; no statistical analysis was performed. Axis scaling was adjusted for visualization. All data were normalized to an endogenous control U24. Relative expression ratios (rERs) were determined using the Schefe’s GED method, which accounts for individual amplification efficiencies of each gene, *p* < 0.05 (*), and *p* < 0.01 (**).

In A549 cells, hsa-let-7a-3p expression was comparable between the control and GFP groups, whereas A549-Twist cells showed significantly higher expression than both groups (Figure 15A). RNA from A549-CSCs was available from only two biological replicates because most of the material was used for small RNA sequencing. Therefore, these data are presented descriptively. Both CSC samples exhibited markedly increased hsa-let-7a-3p expression, with fold changes of 7835 and 172 relative to the control group (Figure 15B), consistent with the RNA-seq results.

In H1975 cells, the overall Kruskal–Wallis test was significant; however, post hoc multiple comparisons did not identify significant pairwise differences among the analyzed groups (Figure 15C). RNA from H1975-CSCs was obtained from a single biological replicate because most of the material was allocated for sequencing. Consequently, these data are also presented descriptively. The H1975-CSC sample showed higher hsa-let-7a-3p expression than the remaining phenotypic groups (Figure 15D), consistent with the expression pattern identified by small RNA sequencing.

### 3.16. Overexpression of hsa-let-7a-3p Was Achieved by Transient Transfection

**Figure 16:**
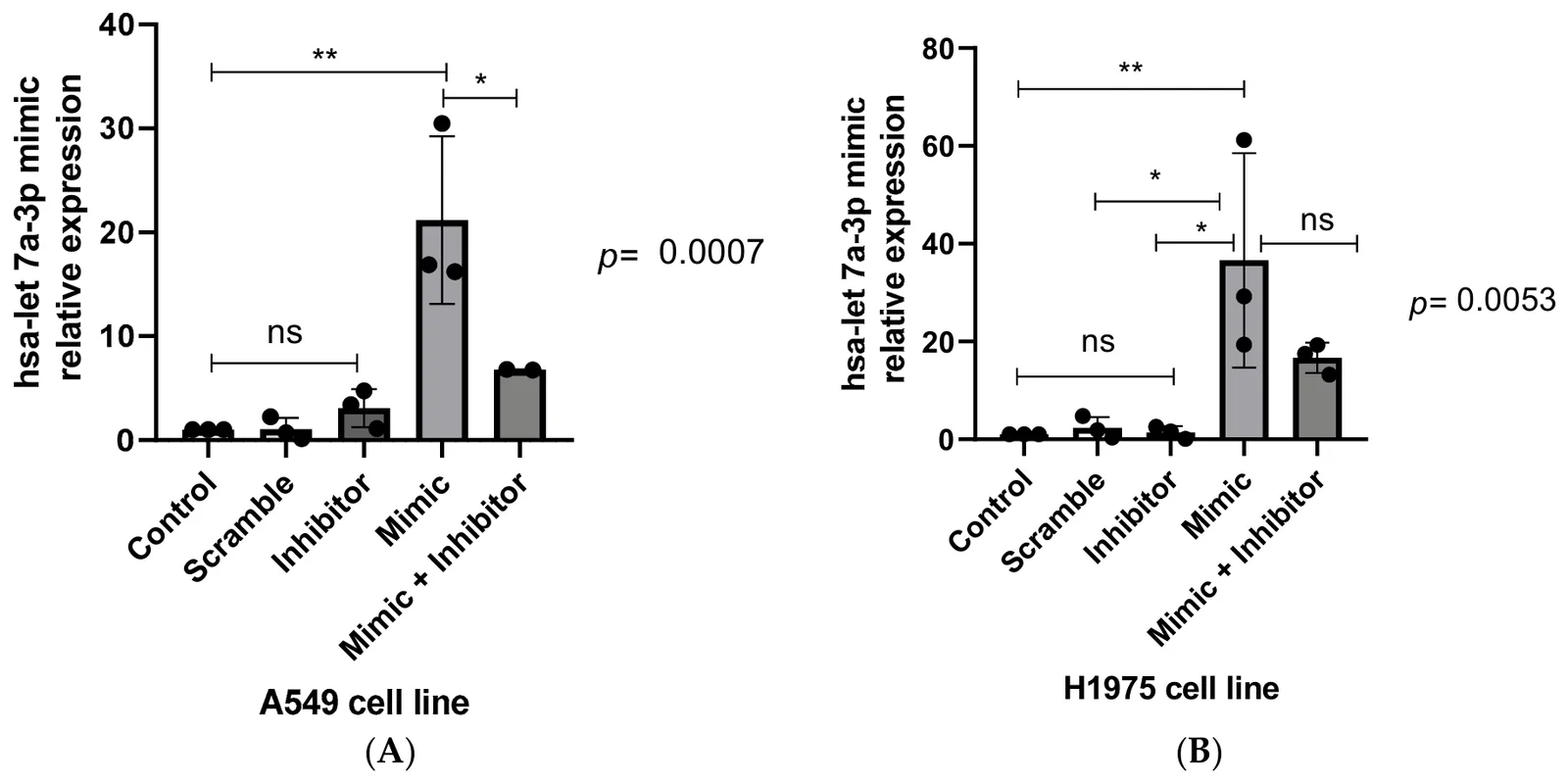
hsa-let-7a-3p mimic was successfully transfected into lung cancer cell lines. qPCR data are presented as mean ± SD/SEM from three biological replicates with technical replicates. One-way ANOVA was used, with statistical significance defined as *p* < 0.05. All data were normalized to the endogenous control U24. Relative expression ratios (rERs) were calculated using the Scheffé’s GED method [15]. (**A**) A549 cell line under different treatment conditions. (**B**) H1975 cell line under different treatment conditions. “ns” indicates no statistically significant difference, *p* < 0.05 (*), and *p* < 0.01 (**).

To investigate the functional role of hsa-let-7a-3p, A549 and H1975 cells were transiently transfected with a hsa-let-7a-3p mimic or a hsa-let-7a-3p inhibitor. Cells transfected with the mimic exhibited significantly higher hsa-let-7a-3p expression than the control and scramble groups in both cell lines (Figure 16), confirming successful overexpression.

To assess the efficiency of miRNA inhibition, cells were co-transfected with the hsa-let-7a-3p mimic and the corresponding inhibitor. Compared with cells transfected with the mimic alone, co-transfected cells exhibited reduced hsa-let-7a-3p expression. This reduction was statistically significant in A549 cells, whereas in H1975 cells a decrease in expression was observed but did not reach statistical significance (Figure 16).

### 3.17. Overexpression of hsa-let-7a-3p Does Not Affect Proliferation in NSCLC Cell Lines

**Figure 17:**
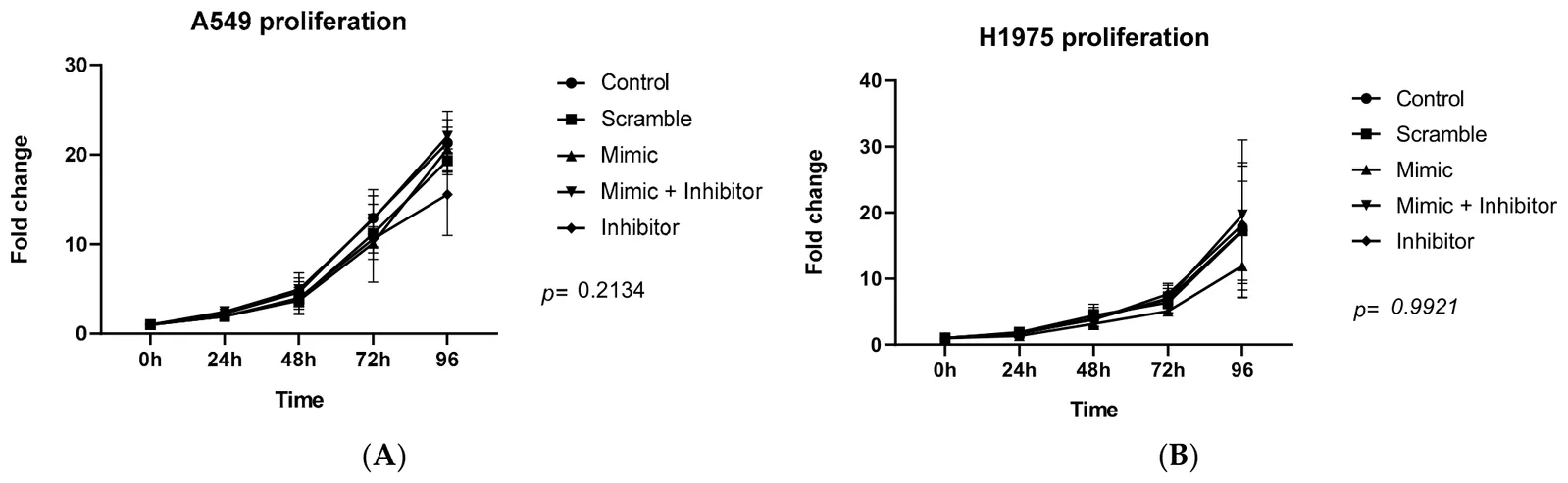
Effect of hsa-let-7a-3p overexpression on the proliferation of lung cancer cell lines. Cell proliferation was evaluated every 24 h for up to 96 h following transfection in A549 (**A**) and H1975 (**B**) cell lines. Data are presented as mean ± SD from three independent biological replicates, each performed with technical replicates. Statistical analysis was performed using two-way ANOVA, with significance defined as *p* < 0.05. The data were processed and expressed as fold change relative to the mean value of the control at time 0.

Following transient transfection with the hsa-let-7a-3p mimic, cell proliferation was evaluated using the CyQUANT^®^ Direct Proliferation Assay. No significant differences in proliferation were observed between hsa-let-7a-3p-overexpressing cells and their corresponding control groups in either NSCLC cell line (Figure 17), indicating that hsa-let-7a-3p overexpression did not affect cell proliferation under the experimental conditions evaluated.

### 3.18. hsa-let-7a-3p Overexpression Showed a Trend Toward Reduced Invasive Capacity in NSCLC Cell Lines

**Figure 18:**
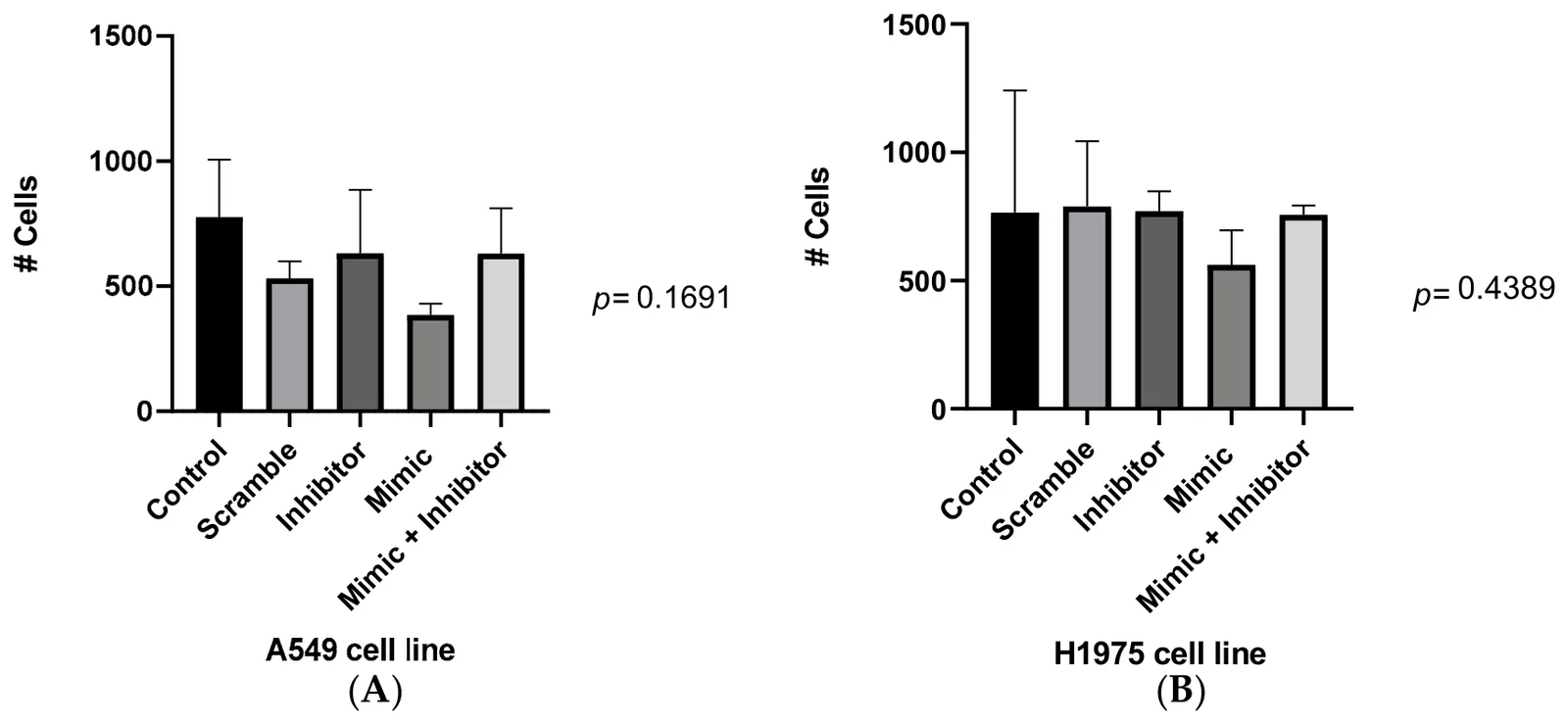
Effect of hsa-let-7a-3p modulation on the invasive capacity of lung cancer cell lines. Cell invasion was assessed in A549 (**A**) and H1975 (**B**) cell lines following transfection with the hsa-let-7a-3p mimic, inhibitor, or their corresponding controls. The *Y*-axis represents the number of invading cells (# cells), and the *X*-axis represents the different experimental conditions. Data are presented as the mean ± SD of three independent biological replicates, each with technical replicates. Statistical analysis was performed using one-way ANOVA, with statistical significance defined as *p* < 0.05.

Following transient transfection with the hsa-let-7a-3p mimic, invasive capacity was evaluated using the Abcam Cell Invasion Assay. Although no statistically significant differences were observed between hsa-let-7a-3p-overexpressing cells and the corresponding control groups in either NSCLC cell line (one-way ANOVA, *p* > 0.05), lower invasion values were consistently observed following hsa-let-7a-3p overexpression (Figure 18). Co-transfection with the hsa-let-7a-3p inhibitor increased invasion relative to cells transfected with the mimic alone, although these differences were also not statistically significant.

### 3.19. hsa-let-7a-3p Overexpression Reduces Clonogenic Capacity in NSCLC Cell Lines

Finally, clonogenic capacity was evaluated following transient transfection with the hsa-let-7a-3p mimic. Cells were cultured for 10 days, after which colonies containing ≥50 cells were counted.

**Figure 19:**
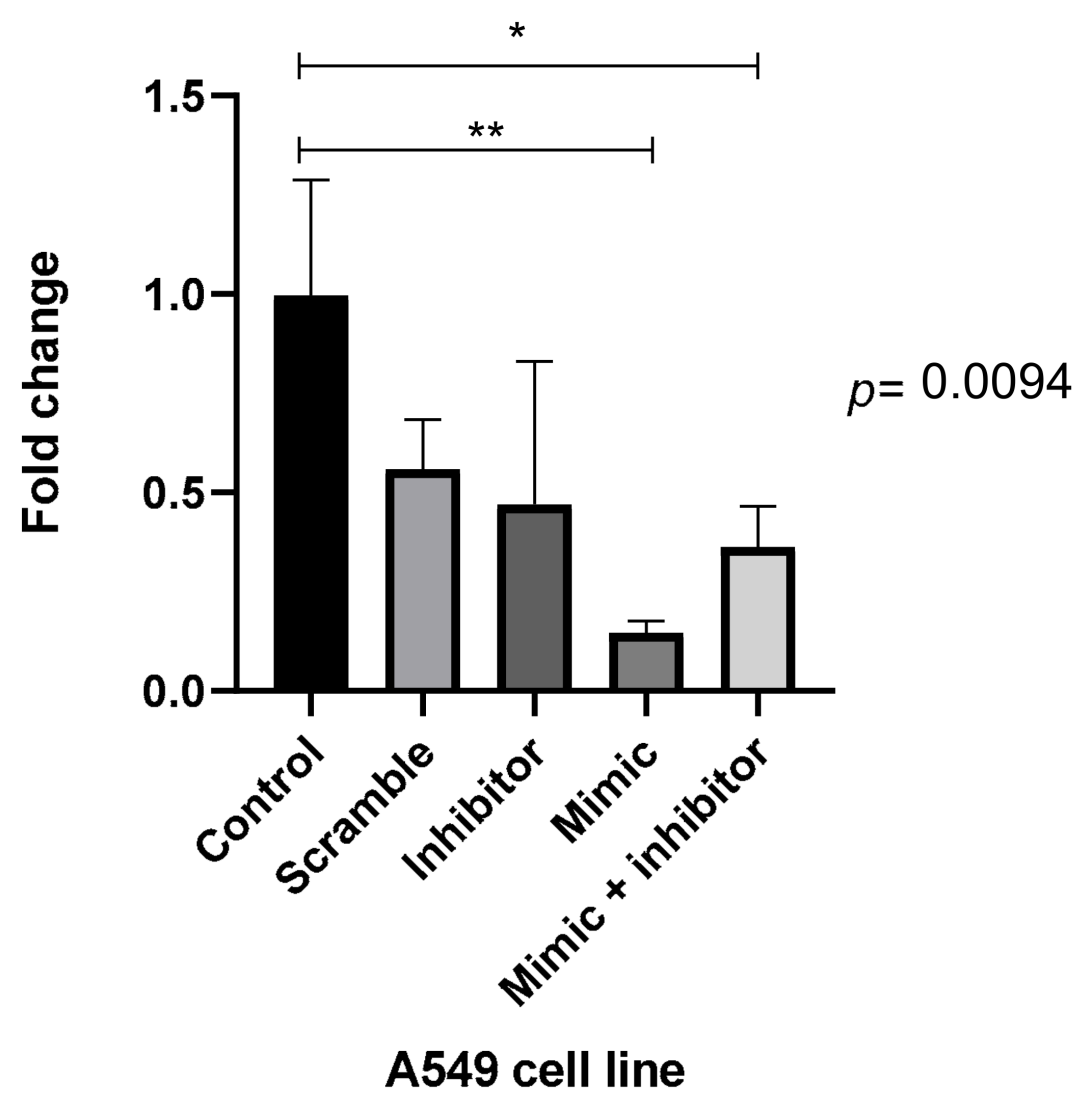
Effect of hsa-let-7a-3p overexpression on the clonogenic capacity of the A549 cell line. Cells overexpressing the miRNA showed a reduction in colony formation compared with the control condition. Data were normalized to the control and expressed as fold change (*Y*-axis). Results are presented as mean ± SD from three independent biological replicates with technical replicates. Statistical analysis was performed using one-way ANOVA, with significance defined as *p* < 0.05. *p* < 0.05 (*), and *p* < 0.01 (**).

In A549 cells, hsa-let-7a-3p overexpression significantly reduced colony formation compared with the control groups (*p* < 0.01) (Figure 19). Co-transfection with the hsa-let-7a-3p inhibitor increased the number of colonies relative to cells transfected with the mimic alone.

## 4. Discussion

The induction of epithelial–mesenchymal transition (EMT) is widely used to enrich cancer stem cell (CSC)-like populations in experimental cancer models [30,31,32]. In the present study, CRISPR/dCas9-mediated activation of TWIST successfully induced an EMT-like phenotype in A549 cells but failed to promote TWIST overexpression in H1975 cells. This finding highlights the context-dependent nature of EMT induction and suggests that the response to TWIST activation is strongly influenced by the molecular characteristics of individual NSCLC cell lines.

A549 and H1975 were selected as complementary NSCLC models because they represent distinct molecular backgrounds. A549 cells harbor KRAS mutations and are extensively used to investigate EMT and tumor plasticity, whereas H1975 cells carry the clinically relevant EGFR L858R/T790M mutations, which are associated with therapeutic resistance, epithelial–mesenchymal plasticity, and stem-like properties [33]. The different responses observed following TWIST activation therefore emphasize the influence of the genetic background on EMT induction. 

The inability to induce TWIST expression in H1975 cells is unlikely to reflect a technical limitation of the CRISPR/dCas9 system, which can vary widely across different cellular contexts, depending on factors such as the delivery method and intrinsic properties of each cell line that influence editing outcomes [34]. Previous studies have demonstrated efficient CRISPR-mediated gene editing in both A549 and H1975 cells using different delivery strategies [35,36], supporting the viability of genome engineering in these models. Instead, our findings suggest that intrinsic regulatory mechanisms specific to H1975 cells may limit TWIST activation under the experimental conditions used. Consistent with this interpretation, Pallier et al. reported that EGFR-mutant lung cancer cell lines lacking detectable TWIST expression, including H1975, failed to undergo EMT following EGF stimulation, whereas EMT induction occurred only in cells expressing TWIST [33]. These observations indicate that the regulation of TWIST in EGFR-mutant NSCLC is highly context dependent and may involve additional molecular or epigenetic mechanisms beyond receptor signaling.

Although the mechanisms responsible for the absence of TWIST expression in H1975 remain unclear, our results reinforce the concept that EMT induction is not uniform across NSCLC models. Instead, the acquisition of mesenchymal traits appears to depend on intrinsic molecular and genetic characteristics, highlighting the importance of considering tumor heterogeneity when studying EMT-associated regulatory programs.

Characterization of the transduced populations demonstrated that TWIST activation in A549 cells induced the concurrent expression of epithelial and mesenchymal markers, consistent with a hybrid epithelial–mesenchymal (E/M) phenotype rather than a complete EMT. Although mesenchymal markers, including N-cadherin, fibronectin, and ZEB1, were upregulated, epithelial markers such as E-cadherin, cytokeratin, and occludin remained expressed (Figure 1). These findings support the current view that EMT is not a binary process but rather a dynamic continuum in which tumor cells adopt intermediate phenotypic states with enhanced plasticity [37,38,39].

Hybrid E/M states have been consistently associated with increased stem-like properties, tumor-initiating capacity, and metastatic competence [37]. Consistent with this concept, A549-TWIST cells also exhibited increased expression of CSC-associated markers, including ALDH, ABCG2, and EpCAM (Figure 2), supporting the notion that partial EMT may facilitate the acquisition of stem-like characteristics rather than complete mesenchymal differentiation. These findings further support the close functional relationship between epithelial plasticity and CSC biology.

In contrast, H1975 cells did not respond to TWIST activation (Figure 1), which is consistent with previous reports describing this cell line as intrinsically displaying a hybrid E/M phenotype characterized by the simultaneous expression of epithelial and mesenchymal markers [40]. Interestingly, we observed that lentiviral GFP transduction alone altered the expression of several EMT-related markers, suggesting that the transduction procedure itself may influence gene expression independently of TWIST activation. This observation highlights the importance of including appropriate transduction controls when evaluating EMT-associated transcriptional changes.

Despite the absence of detectable TWIST overexpression in H1975 cells, CD133^+^ CSC-enriched populations were successfully generated under stem cell culture conditions. This finding reinforces the concept that CSC acquisition is not exclusively dependent on TWIST-mediated EMT but may arise through multiple mechanisms of tumor plasticity. Increasing evidence indicates that cancer cells can reversibly transition between non-stem and stem-like states in response to intrinsic regulatory programs and microenvironmental cues [41,42,43]. Therefore, our results further support the dynamic relationship between EMT, cellular plasticity, and CSC biology in NSCLC. Where EMT facilitated CSC enrichment in A549 cells but was not an absolute prerequisite for CSC generation, as demonstrated in the H1975 cell line (Figure 3). Instead, different biological mechanisms may converge on common post-transcriptional regulatory networks associated with stemness.

Identifying the regulatory mechanisms underlying these phenotypes has become a major focus of cancer research. Among these mechanisms, miRNAs have emerged as key post-transcriptional regulators capable of coordinately modulating multiple target genes involved in EMT, stemness, and tumor progression [11,44,45,46,47].

To identify miRNAs specifically associated with CSC acquisition while minimizing changes attributable to lentiviral transduction, GFP expression, or TWIST activation alone, we implemented a sequential filtering strategy integrating differential expression analyses across multiple experimental conditions and two molecularly distinct NSCLC cell lines. This approach identified a conserved CSC-associated miRNA signature, suggesting that, despite their distinct genetic backgrounds, both models converge on common post-transcriptional regulatory programs. Furthermore, pathway enrichment and miRNA–target interaction network analyses demonstrated that these miRNA signatures coordinately regulate signaling pathways involved in EMT, Wnt, TGF-β, mTOR, focal adhesion, and cytoskeletal remodeling. This integrative strategy extends beyond the identification of differentially expressed miRNAs by providing mechanistic insight into how groups of miRNAs may collectively regulate the signaling networks that sustain CSC plasticity.

When prioritizing candidate miRNAs, we considered not only differential expression and statistical significance but also their expression abundance. Although fold change is commonly used to rank differentially expressed miRNAs, highly abundant miRNAs may exert substantial biological effects even when exhibiting relatively modest expression changes because they simultaneously regulate numerous target transcripts [48,49,50]. Consistent with this concept, highly expressed miRNAs identified in our study, including hsa-let-7b-5p and hsa-miR-101-3p in CSCs and hsa-miR-181a-5p and hsa-miR-30a-5p during EMT, remained among the most abundant regulatory molecules despite moderate fold changes. Therefore, integrating fold change, adjusted *p*-value, and expression abundance provides a more robust framework for prioritizing biologically relevant candidate miRNAs than relying on a single parameter alone [48,51]. 

Among the commonly dysregulated miRNAs identified in CSC-enriched cells, hsa-miR-424-3p, hsa-miR-455-3p, hsa-miR-130b-5p, hsa-miR-15b-5p, and hsa-let-7b-5p emerged as the most robust downregulated candidates based on their differential expression, statistical significance, and expression abundance. Although hsa-miR-424-3p and hsa-miR-455-3p exhibited the largest reductions in expression, hsa-let-7b-5p showed the highest abundance in both CSC models and was identified as the principal regulatory miRNA within the Wnt, Cell cycle, and TGF-β interaction networks (Figure 6). Members of the let-7 family have been widely implicated in the regulation of stemness, differentiation, and EMT [52,53,54], supporting the biological relevance of our findings and suggesting that reduced hsa-let-7b-5p expression may contribute to the coordinated regulation of signaling pathways involved in CSC maintenance.

A similar pattern was observed among the commonly upregulated miRNAs. Although hsa-miR-1246 displayed the largest fold change, hsa-miR-101-3p represented the major hub within the Wnt and TGF-β interaction networks, whereas hsa-miR-19b-3p showed the highest connectivity within the mTOR signaling pathway (Figure 9). Notably, these miRNAs have previously been associated with EMT, stemness, or NSCLC progression [55,56,57,58,59,60], further supporting the biological relevance of the identified CSC-associated miRNA signature.

An important finding of this study was that, despite identifying distinct sets of upregulated and downregulated miRNAs, both enrichment analyses and interaction networks consistently converged on a limited number of signaling pathways, including Wnt, TGF-β, mTOR, focal adhesion, adherens junction, and regulation of the actin cytoskeleton (Figure 5 and Figure 8) [61,62]. These pathways are functionally interconnected and collectively regulate cellular plasticity, self-renewal, extracellular matrix remodeling, epithelial organization, and migratory behavior [7]. The convergence of multiple miRNAs on the same biological pathways supports the concept that CSC phenotypes are regulated through coordinated post-transcriptional networks rather than by individual miRNAs acting independently.

Comparison of the CSC-associated and TWIST-induced EMT miRNA signatures further demonstrated that, although these phenotypes share several regulatory pathways, they are characterized by distinct patterns of miRNA dysregulation (Supplementary Table S3 and Figure 10, Figure 11, Figure 12 and Figure 13). This distinction is consistent with current evidence indicating that EMT and CSCs represent closely related but biologically distinct processes [38,63], suggesting that specific groups of miRNAs may regulate different stages of cellular plasticity during NSCLC progression.

For instance, the coordinated downregulation of multiple members of the miR-200 family was one of the most relevant findings associated with TWIST-induced EMT (Supplementary Table S3). The miR-200 family is a well-established regulator of epithelial identity through the repression of ZEB1 and ZEB2, and its loss is considered a hallmark of EMT [64,65]. Therefore, its coordinated downregulation further supports the successful induction of a TWIST-driven hybrid EMT program in A549 cells. Moreover, the simultaneous reduction of several highly expressed miRNAs indicates that EMT involves extensive post-transcriptional remodeling instead of the dysregulation of a few individual regulators.

On the other hand, the miRNAs upregulated during TWIST-induced EMT were primarily associated with pathways involved in cellular plasticity, extracellular matrix remodeling, and tumor progression (Figure 12). Interaction network analysis further identified hsa-let-7c-5p as the principal regulatory miRNA within both the TGF-β signaling and regulation of actin cytoskeleton pathways (Figure 13). This miRNA was connected to genes encoding receptors, SMAD family members, and cytoskeletal regulators, including TGFBR1, ACVR2B, BMPR2, SMAD2, SMAD3, SMAD4, SMAD5, ROCK1, ROCK2, THBS1, CREBBP, and EP300, suggesting that hsa-let-7c-5p may coordinately regulate canonical TGF-β signaling and cytoskeletal remodeling during EMT.

In contrast, the focal adhesion network exhibited a more distributed regulatory architecture, with multiple miRNAs converging on genes involved in integrin signaling (ITGA5 and ITGB1), focal adhesion dynamics (PXN, VCL, ACTN1, and ACTN4), receptor tyrosine kinase signaling (EGFR and MET), intracellular signaling (PIK3R1, RAC1, ROCK1, and ROCK2), angiogenic signaling (VEGFA), and cell-cycle regulation (CCND1 and CCND2) (Figure 13). These interactions support a coordinated post-transcriptional regulation of the molecular programs controlling cell adhesion, migration, and cytoskeletal organization during EMT [66,67,68].

Comparison of the EMT- and CSC-associated miRNA signatures further revealed that, although both phenotypes shared several signaling pathways, they were characterized by distinct regulatory networks. Whereas the CSC-associated signature predominantly converged on pathways involved in stemness maintenance, Wnt signaling, and mTOR activation, the EMT-associated signature was more strongly associated with extracellular matrix remodeling, cell adhesion, and cytoskeletal organization. These findings support the concept that EMT and CSC phenotypes are governed by overlapping yet distinct miRNA-mediated regulatory programs, reflecting different stages of tumor cell plasticity.

Based on the identified CSC-associated miRNA signature, hsa-let-7a-3p was selected for functional validation because it showed consistent upregulation across CSC-enriched populations derived from both NSCLC cell lines, together with robust fold changes and highly significant adjusted *p*-values (Table 2). Although hsa-let-7a-3p was not among the principal hubs identified in the pathway-specific interaction networks, this likely reflects the degree cutoff (≥5) applied during network construction rather than limited biological relevance. This filtering strategy was implemented to simplify network visualization by retaining only the most highly connected nodes [28]. Consequently, miRNAs with fewer validated interactions within individual pathways may not appear in the final networks despite exhibiting consistent differential expression. Instead, hsa-let-7a-3p was prioritized because its biological role in lung cancer remains poorly characterized despite belonging to the extensively studied let-7 family, making it an attractive candidate for functional investigation.

Members of the let-7 family are widely recognized as tumor-suppressive miRNAs that regulate cell proliferation, self-renewal, epithelial–mesenchymal transition, and metastatic dissemination through the coordinated repression of multiple oncogenic pathways [52,53,54]. Interestingly, hsa-let-7a-3p was consistently overexpressed in CSC-enriched populations from both NSCLC cell lines. Rather than contradicting the established tumor-suppressive role of the let-7 family, this finding suggests that individual mature let-7 strands may exert distinct context-dependent regulatory functions. Therefore, the biological role of hsa-let-7a-3p cannot be inferred solely from the functions attributed to other members of the family and requires direct functional characterization in NSCLC.

Although few studies have specifically investigated hsa-let-7a-3p, available evidence supports a tumor-suppressive role in other malignancies. Bahojb and colleagues and Mozammel and collaborators demonstrated that hsa-let-7a-3p overexpression inhibited cell-cycle progression and induced apoptosis in U87MG glioblastoma and HCT-116 colorectal cancer cells, respectively. In addition, hsa-let-7a-3p reduced cell migration through the regulation of matrix metalloproteinases MMP-2 and MMP-9, two important mediators of tumor invasion and metastasis [69,70]. However, to our knowledge, its biological function has not previously been investigated in lung cancer.

Bioinformatic analyses further supported the potential involvement of hsa-let-7a-3p in CSC biology. Target genes were significantly enriched in pathways related to stem-cell pluripotency, TGF-β signaling, Hippo signaling, AMPK signaling, focal adhesion, endocytosis, ATP-dependent chromatin remodeling, and other cancer-associated processes (Figure 14 and Table 3). These pathways regulate epithelial plasticity, self-renewal, metabolic adaptation, and metastatic dissemination [71,72,73,74], suggesting that hsa-let-7a-3p may coordinately modulate several biological programs relevant to CSC maintenance rather than acting on a single signaling pathway.

Consistent with these bioinformatic predictions, functional validation demonstrated that hsa-let-7a-3p primarily affected phenotypes associated with CSC behavior rather than cell proliferation. Overexpression of hsa-let-7a-3p did not significantly alter proliferation in either NSCLC cell line (Figure 17), indicating that this miRNA is unlikely to regulate short-term proliferative capacity under standard culture conditions. This observation is consistent with the current understanding that CSC biology depends more on cellular plasticity and adaptability than on sustained proliferation, which also contributes to the intrinsic resistance of these cells to conventional chemotherapeutic agents targeting rapidly dividing populations [75,76].

In contrast, hsa-let-7a-3p influenced biological processes associated with tumor aggressiveness. Although overexpression produced only a non-significant reduction in invasion, both NSCLC cell lines exhibited the same directional trend, and this effect was partially reversed following co-transfection with the miRNA inhibitor (Figure 18). Similar anti-invasive effects have been reported in glioblastoma and colorectal cancer models [69,70], supporting a conserved role of hsa-let-7a-3p in regulating invasion-associated mechanisms across different tumor types.

The most pronounced functional effect was observed in the clonogenic assay. Overexpression of hsa-let-7a-3p significantly reduced colony formation in A549 cells (Figure 19), indicating diminished long-term self-renewal capacity. Because clonogenic growth is widely considered a functional surrogate of CSC self-renewal [75], these findings suggest that hsa-let-7a-3p preferentially regulates stem-like properties rather than proliferative activity. Although clonogenic assays were performed only in A549 cells due to the robust and reproducible colony-forming capacity of this model, the observed phenotype is consistent with the enrichment analyses predicting the involvement of hsa-let-7a-3p in pathways controlling stem-cell maintenance.

Collectively, the bioinformatic and functional data indicate that hsa-let-7a-3p acts as a context-dependent regulator of CSC biology in NSCLC. Rather than functioning as a central driver of aggressive phenotypes. Although hsa-let-7a-3p was consistently overexpressed in CSC-enriched populations, its functional overexpression reduced clonogenic capacity and showed a tendency to decrease invasion without affecting proliferation. One possible explanation is that hsa-let-7a-3p participates in compensatory feedback mechanisms that maintain the balance between stemness, plasticity, and survival in CSCs, preventing excessive activation of oncogenic signaling pathways. Such regulatory circuits may contribute to preserving phenotypic equilibrium rather than simply promoting tumor aggressiveness. Moreover, because dormant CSCs are characterized by low proliferative activity together with long-term survival and therapy resistance [77,78,79], the expression pattern observed here raises the possibility that hsa-let-7a-3p could also participate in regulatory programs associated with dormant CSC states. Although these hypotheses require direct experimental validation, they provide a plausible biological framework to explain the context-dependent behavior of hsa-let-7a-3p observed in this study.

## 5. Conclusions

This study provides an integrative characterization of the miRNA regulatory programs associated with epithelial–mesenchymal transition (EMT) and cancer stem cell (CSC) enrichment in NSCLC. By combining differential expression analysis, pathway enrichment, and miRNA–target interaction networks, we identified conserved miRNA signatures shared by CSCs derived from two molecularly distinct lung cancer cell lines and demonstrated that these signatures converge on signaling pathways governing epithelial plasticity, stemness, and tumor progression.

Functional validation identified hsa-let-7a-3p as a context-dependent regulator of CSC biology. Although consistently overexpressed in CSC-enriched populations, hsa-let-7a-3p primarily affected clonogenicity and showed a tendency to reduce invasion without altering cell proliferation, suggesting that its function extends beyond conventional tumor-suppressive or oncogenic roles. Together with the bioinformatic analyses, these findings support the hypothesis that hsa-let-7a-3p contributes to the fine regulation of signaling networks controlling CSC plasticity and may participate in regulatory programs associated with CSC maintenance and dormancy.

Overall, this work expands the current understanding of post-transcriptional regulation in NSCLC and provides a framework for identifying miRNA signature-mediated regulatory networks involved in CSC biology. These findings establish a foundation for future mechanistic studies aimed at elucidating the functional roles of additional CSC-associated miRNAs and evaluating their potential as diagnostic biomarkers targets in lung cancer.

## 6. Limitations

Several limitations of this study should be acknowledged. Functional analyses of hsa-let-7a-3p were limited to in vitro models and a restricted set of CSC-associated phenotypes. Additionally, although bioinformatic analyses predicted the involvement of hsa-let-7a-3p in key CSC-related signaling pathways, direct validation of specific target genes was not addressed. Future studies should focus on validating these targets, exploring the role of hsa-let-7a-3p in vivo, and evaluating its interaction with EMT- and stemness-related pathways. Additionally, exploring the regulation and function of hsa-let-7a-3p across different lung cancer subtypes and treatment conditions may provide further insight into its potential role as a biomarker or therapeutic modulator in CSC-driven tumor progression.

## Figures and Tables

**Table 1:** List of the miRNAs downregulated of CD133^+^ CSC-enriched cells derived from both cell lines A549 and H1975. LogFC = logarithm of fold change.

miRNAs	LogFC A549-CSC	LogFC H1975-CSC	P-Adj A549-CSC	P-Adj H1975-CSC	BaseMean A549-CSC	BaseMean H1975-Csc
**hsa-miR-424-3p**	−3.805	−3.045	2.00 × 10^−20^	4.90 × 10^−13^	837.4	267.78
**hsa-miR-130b-5p**	−3.268	−2.200	8.60 × 10^−9^	1.70 × 10^−6^	142.78	880.99
**hsa-miR-455-3p**	−2.985	−3.475	1.20 × 10^−15^	1.30 × 10^−16^	965.53	9007.81
**hsa-miR-15b-5p**	−2.913	−2.489	1.80 × 10^−10^	1.40 × 10^−5^	893.67	2657.71
**hsa-miR-181b-5p,1**	−2.447	−1.595	3.70 × 10^−8^	9.00 × 10^−4^	7122.69	2657.71
**hsa-miR-181b-5p**	−2.451	−1.519	5.60 × 10^−8^	1.10 × 10^−3^	6691.51	2657.71
**hsa-miR-196b-5p**	−2.354	−2.703	6.60 × 10^−7^	3.50 × 10^−7^	370.67	63.03
**hsa-let-7b-5p**	−2.089	−2.285	7.80 × 10^−6^	1.60 × 10^−7^	21,320.05	93,300.3
**hsa-miR-505-3p**	−1.955	−3.157	3.00 × 10^−5^	4.30 × 10^−13^	153.55	9007.81
**hsa-miR-181d-5p**	−1.758	−3.394	3.30 × 10^−6^	1.10 × 10^−12^	625.66	2657.71
**hsa-miR-345-5p**	−1.740	−1.725	1.00 × 10^−4^	1.30 × 10^−3^	258.32	267.78
**hsa-miR-34a-5p**	−1.676	−1.544	2.60 × 10^−4^	2.10 × 10^−2^	1031.44	267.78
**hsa-miR-10527-5p**	−1.573	−2.658	8.00 × 10^−3^	5.00 × 10^−4^	56.97	53.62

**Table 2:** List of the miRNAs upregulated of CD133^+^ CSC-enriched cells derived from both cell lines A549 and H1975. LogFC = logarithm of fold change.

miRNAs	LogFC A549-CSC	LogFC H1975-CSC	P-Adj A549-CSC	P-Adj H1975-CSC	BaseMean A549-CSC	BaseMean H1975-Csc
**hsa-miR-374a-3p**	6.143	5.033	1.11 × 10^−23^	2.52 × 10^−8^	141.19	68.85
**hsa-miR-32-5p**	6.012	5.021	2.49 × 10^−17^	2.18 × 10^−15^	95.18	88.71
**hsa-miR-582-3p**	5.898	3.956	1.45 × 10^−37^	2.70 × 10^−9^	858.28	229.8
**hsa-miR-374a-5p**	5.571	4.302	5.21 × 10^−30^	3.18 × 10^−10^	214.87	191.73
**hsa-miR-19b-3p**	5.543	3.654	6.34 × 10^−15^	1.05 × 10^−5^	62.2	104.12
**hsa-miR-19b-3p,1**	5.531	3.711	6.69 × 10^−15^	5.86 × 10^−6^	61.72	104.47
**hsa-let-7a-3p**	4.776	4.006	2.49 × 10^−17^	1.78 × 10^−15^	75.63	165.02
**hsa-let-7a-3p,1**	4.762	4.006	2.49 × 10^−17^	1.78 × 10^−15^	75.47	165.02
**hsa-miR-1246**	4.474	7.078	4.85 × 10^−22^	5.47 × 10^−56^	263.67	799.72
**hsa-miR-101-3p,1**	3.701	3.600	6.63 × 10^−18^	4.21 × 10^−10^	1186.13	2208.64
**hsa-miR-101-3p**	3.694	3.629	1.03 × 10^−17^	1.35 × 10^−10^	1155.34	2123.71
**hsa-miR-340-5p**	3.451	1.742	1.82 × 10^−12^	1.09 × 10^−2^	202.71	88.71
**hsa-miR-100-5p**	3.129	1.772	2.06 × 10^−6^	2.19 × 10^−4^	107,750.15	68.85

**Table 3:** Enrichment analysis of genes regulated by hsa-let-7a-3p using DIANA-miRPath v4.0.

*Term Name*	*Term Genes*	*miRNAs (n)*	*miRNA Names*	*Merged p-Value*	*Merged FDR*
*Pathways in cancer*	555	1	hsa-let-7a-3p	0.0000307	0.000307
*Hippo signaling pathway*	164	1	hsa-let-7a-3p	0.000229	0.00044
*Tight junction*	182	1	hsa-let-7a-3p	0.00014	0.00044
*Regulation of actin cytoskeleton*	224	1	hsa-let-7a-3p	0.000264	0.00044
*Human papillomavirus infection*	406	1	hsa-let-7a-3p	0.000182	0.00044
*Small cell lung cancer*	100	1	hsa-let-7a-3p	0.0000943	0.00044
*PI3K-Akt signaling pathway*	372	1	hsa-let-7a-3p	0.00053	0.00066
*Focal adhesion*	213	1	hsa-let-7a-3p	0.000594	0.00066
*MicroRNAs in cancer*	334	1	hsa-let-7a-3p	0.000465	0.00066
*ECM-receptor interaction*	104	1	hsa-let-7a-3p	0.000675	0.000675

## Data Availability

The sequencing data generated in this study have been deposited in the NCBI BioProject database under accession number PRJNA1404308. All other data supporting the findings of this study are included within the article and its Supplementary Materials.

## Supplementary Materials

The following supporting information can be downloaded at: https://www.mdpi.com/article/10.3390/biomedicines14081737/s1, Figure S1: Optimization of lentiviral transduction efficiency in NSCLC cell lines; Figure S2: Determination of antibiotic concentrations required to eliminate non-transduced cells; Figure S3: Assessment of proliferation over time in both A549 and H1975 cell line. Table S1: Primers sequence; Table S2: Profile of miRNAs Exclusively Dysregulated in the A549-GFP Phenotype; Table S3: Profile of miRNAs Exclusively Dysregulated in the A549-Twist Phenotype; Table S4: Profile of miRNAs Exclusively Dysregulated in CSCs derived from A549 Phenotype; Table S5: Profile of miRNAs Exclusively Dysregulated in H1975-GFP Phenotype; Table S6: Profile of miRNAs Exclusively Dysregulated in H1975-Twits Phenotype; Table S7: Profile of miRNAs Exclusively Dysregulated in CSCs derived from H1975 Phenotype; Table S8: Targets of hsa let 7a-3; Table S9: KEEG Pathways.

## Abbreviations

ABCG2	ATP-binding cassette transporter G2
ACVR2B	Activin A receptor type 2B
ACTN1	Alpha-actinin 1
ACTN4	Alpha-actinin 4
ALDH	Aldehyde dehydrogenase
bFGF	Basic fibroblast growth factor
BMPR2	Bone morphogenetic protein receptor type 2
CCND1	Cyclin D1
CCND2	Cyclin D2
CD133	Cluster of Differentiation 133
CREBBP	CREB-binding protein
CSCs	CD133^+^ cancer stem cell-enriched cells
dCas9	Deactivated Cas9
EGF	Epidermal growth factor
EGFR	Epidermal growth factor receptor
EMT	Epithelial-to-mesenchymal transition
EpCAM	Epithelial cell adhesion molecule
EP300	E1A-binding protein p300
H1975	NCI-H1975 cell line
ITS	Insulin-Transferrin-Selenium
ITGA5	Integrin subunit alpha 5
ITGB1	Integrin subunit beta 1
MAPK	Mitogen-activated protein kinase
MET	MET proto-oncogene, receptor tyrosine kinase
miRNAs	MicroRNAs
MMP-2	Matrix metalloproteinase-2
MMP-9	Matrix metalloproteinase-9
MOI	Multiplicity of infection
mTOR	Mechanistic target of rapamycin
NSCLC	Non-small cell lung cancer
PIK3R1	Phosphoinositide 3-kinase regulatory subunit 1
PXN	Paxillin
RAC1	Rac family small GTPase 1
rER	Relative expression ratio
ROCK1	Rho-associated coiled-coil containing protein kinase 1
ROCK2	Rho-associated coiled-coil containing protein kinase 2
SAM	Synergistic activation mediator
SMAD2	SMAD family member 2
SMAD3	SMAD family member 3
SMAD4	SMAD family member 4
SMAD5	SMAD family member 5
TGF-β	Transforming growth factor beta
TGFBR1	Transforming growth factor beta receptor 1
THBS1	Thrombospondin 1
VCL	Vinculin
VEGFA	Vascular endothelial growth factor A
WNT	Wingless/Integrated
ZEB1	Zinc finger E-box-binding homeobox 1
