# Supplementary material for: Identification of a CSC-Associated miRNA Signature in NSCLC and Functional Characterization of hsa-let-7a-3p
Source: Biomedicines. 2026 Jul 31;14(8):1737. doi: 10.3390/biomedicines14081737 (PMC13509304; doi:10.3390/biomedicines14081737)
Supplement: Supplementary file 1 [file biomedicines-14-01737-s001.zip › Supplementary figures (4) (1).pdf]

Supplementary figures

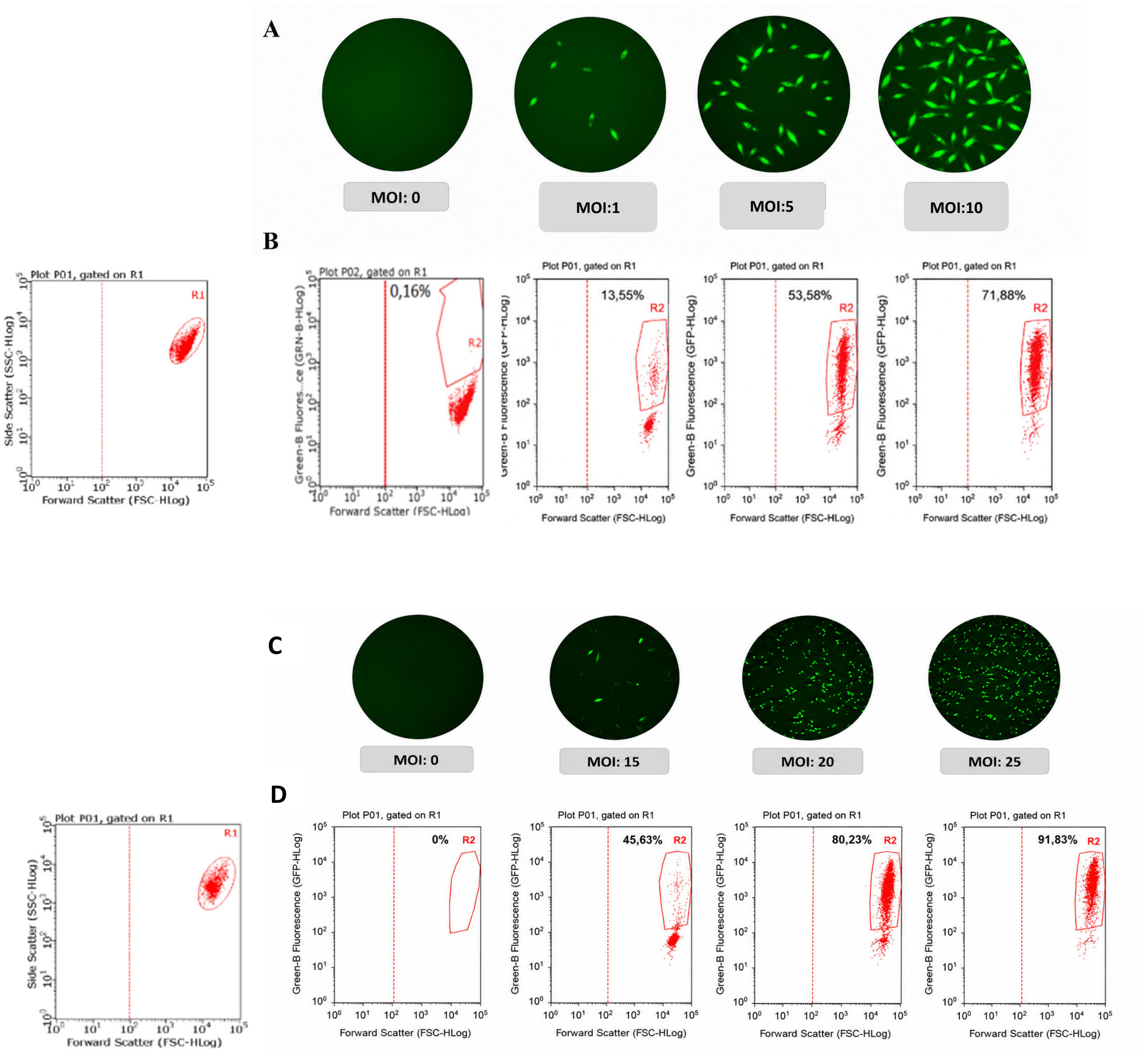

Figure S1. Optimization of lentiviral transduction efficiency in NSCLC cell lines. *A549 and NCI-H1975 cells were transduced with copGFP lentiviral particles (sc-108084) using different multiplicities of infection (MOIs). Representative fluorescence microscopy images show GFP expression at each MOI, allowing visual assessment of transduction efficiency (A, C). Flow cytometry was performed to quantify the percentage of GFP-positive events (B, D). In the dot plots, R1 represents the total viable cell population, whereas R2 indicates the GFP-positive cell population within the R1 gate. Based on both fluorescence microscopy and flow*

cytometry analyses, an MOI of 10 was selected for A549 cells, resulting in 71.88% GFP-positive cells, whereas an MOI of 20 was selected for NCI-H1975 cells, achieving a transduction efficiency of 89.23%.

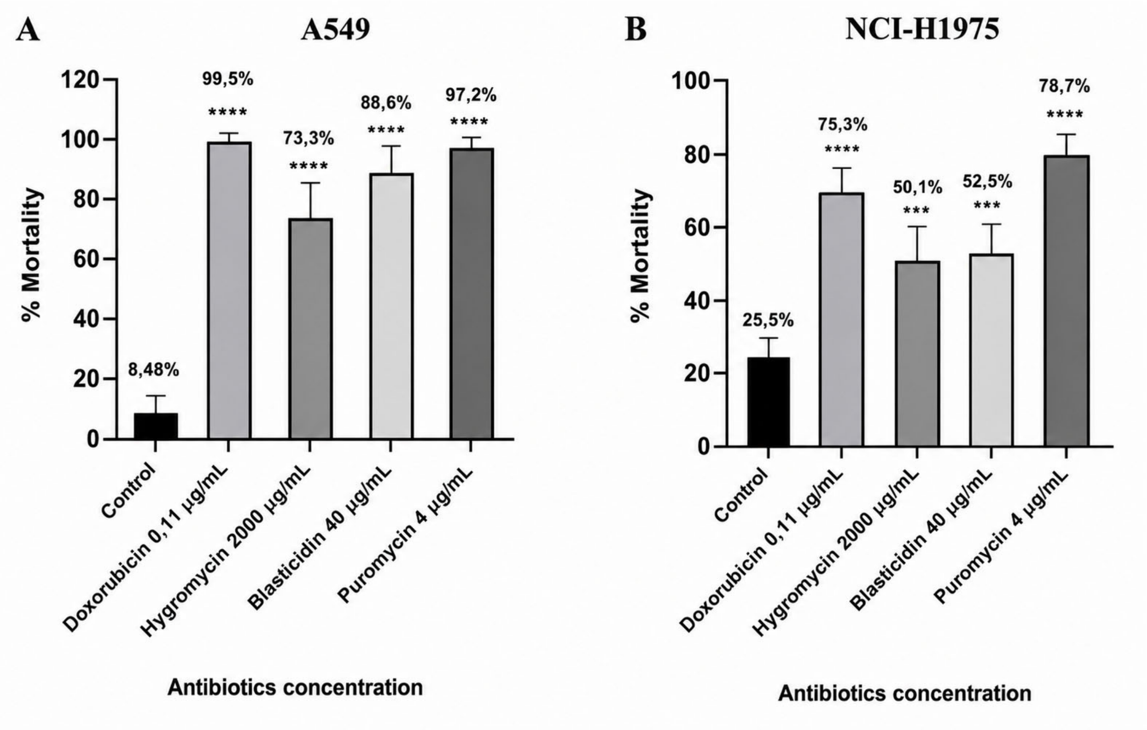

Figure S2. Determination of antibiotic concentrations required to eliminate non-transduced cells. *To identify the optimal concentration for each antibiotic, a dose–response (calibration) curve was performed for both cell lines. The goal was to determine the minimum concentration capable of inducing more than 70% cell death. The selected concentrations are shown for (A) A549 and (B) NCI-H1975 cells. Doxorubicin was used as a positive control for cell death. Cell viability was assessed by flow cytometry using propidium iodide (PI) staining. Results represent three biological replicates with their corresponding technical replicates. Statistical analysis was performed using one-way ANOVA, with significance defined as $p < 0.05$.*

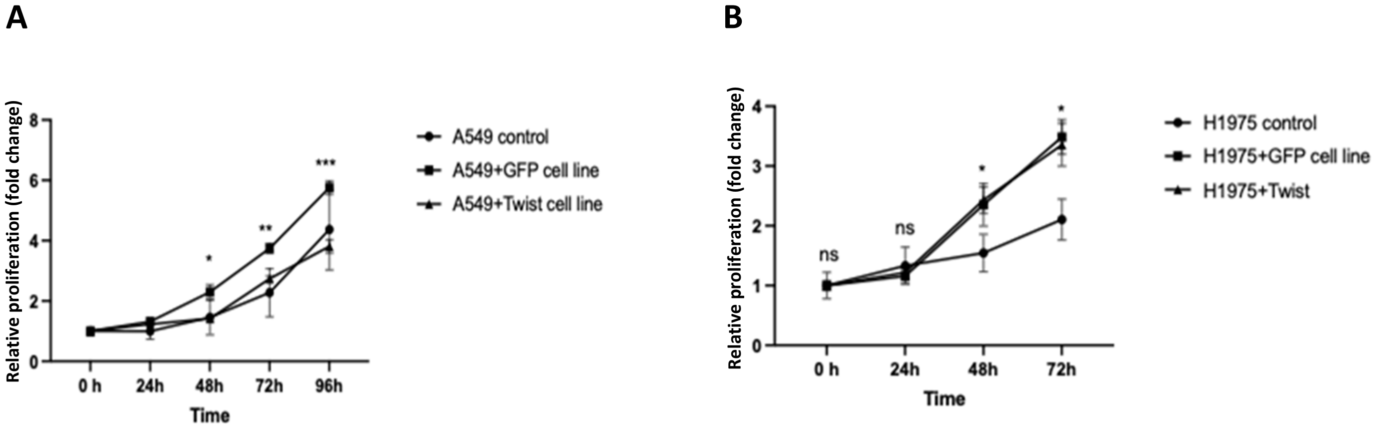

Figure S3. Assessment of proliferation over time in both A549 and H1975 cell line

Each cell condition was evaluated using the MTT assay. A total of 2,500 cells were seeded per well in a 96-well plate, and measurements were taken every 24 hours up to 96 hours. Results represent three biological replicates with their corresponding technical replicates. The absorbances data were normalized to the control condition for each cell line and presented as fold change. Statistical analysis was performed using two-way ANOVA, with significance defined as $p < 0.05$.
